# Purification and characterization of antibacterial surfactin isoforms produced by *Bacillus velezensis* SK

**DOI:** 10.1186/s13568-022-01348-3

**Published:** 2022-01-27

**Authors:** Sagar S. Barale, Savaliram G. Ghane, Kailas D. Sonawane

**Affiliations:** 1 Department of Microbiology, Shivaji University, Kolhapur, 416004 Maharashtra India; 2 Structural Bioinformatics Unit, Department of Biochemistry, Shivaji University, Kolhapur, 416004 Maharashtra India; 3 Department of Botany, Shivaji University, Kolhapur, 416004 Maharashtra India

**Keywords:** Antibiotic-resistance, Antimicrobial peptides, *B. velezensis* SK, Lipopeptide, RP-HPLC, LC–ESI–MS

## Abstract

*Bacillus velezensis* SK having broad-spectrum antimicrobial activity has been isolated from soil. The efficient extraction of antimicrobial compounds produced in various mediums has been done using Diaion HP-20 resin. Further, characterization of an antimicrobial compound by TLC, FTIR, *in-situ* bioautography analysis revealed the presence of cyclic lipopeptides, which is then purified by the combination of silica gel, size exclusion, dual gradient, and RP-HPLC chromatography techniques. Growth kinetic studies showed that *Bacillus velezensis* SK produces a mixture of lipopeptides (1.33 gL^−1^). The lipopeptide exhibits good pH (2–10) and temperature stability up to 80 °C. LC–ESI–MS analysis of partially purified lipopeptide identified variant of surfactin, further analysis of purified chromatographic fractions revealed the occurrence of most abundant C_15_-surfactin homologues (m/z 1036.72 Da). The isolated surfactin exhibits good antimicrobial activity (1600 AU/ml) against drug-resistant food-born *B. cereus* and human pathogen *Staphylococcus aureus*. Hence, identified strain *B. velezensis* SK and its potent antibacterial surfactin lipopeptide could be used in various food and biomedical applications.

**Supplementary Information:**

The online version contains supplementary material available at 10.1186/s13568-022-01348-3.

## Introduction

Antibiotic resistance is an excellent example of microbial acclimatization towards conventional antibiotics (Wright 2007). Improper and unregulated use of antibiotics in the hospital environment, poultry industries, fish farming, and food preservation resulted in the spread and rise into antibiotic resistance phenotype (Hassan et al. 2012; Nadaf et al. 2018; Parulekar and Sonawane 2018a, b; Parulekar et al. 2019a; Wright 2007). The spread of antibiotic resistance in opportunistic pathogens associated with nosocomial infections such as *Escherichia coli*, *Staphylococcus aureus,* and *Pseudomonas aeruginosa* limits the therapeutic options, further resistance heighten due to pathogen in biofilm (Wright 2007). The scarcity of new antimicrobial agents for the treatment of drug-resistant microorganisms further reasoned for the spread of antibiotic resistance and emergence of multidrug-resistant strains (MDR), which has threatened human health worldwide (Alekshun and Levy 2007; Nadaf et al. 2018; Wright 2007). Thus, new effective and safe antimicrobial agents are much needed to control emerging drug-resistant microorganisms (Parulekar and Sonawane 2018a, b; Sumi et al. 2014; Wright 2007). Such health problems led down to a search for natural habitat for new potent and safe antimicrobial agents producing microorganisms from the natural environment (Chopra et al. 2014; Yang et al. 2016). The natural environment remains a promising reservoir of microorganisms, where they constantly evolve to sustain in the competitive and dynamic environment with the production of new antimicrobial agents such as antimicrobial peptides (AMPs) and lipopeptides (Alekshun and Levy 2007; Nadaf et al. 2018; Roy et al. 2015; Waghmare et al. 2019).

Among the AMPs, microbial lipopeptides received considerable attention due to their structural and functional diversity with respect to size, the spectrum of antimicrobial, antifungal, and antiviral activity with low level of resistance and low toxicity (Ongena and Jacques 2008; Kulimushi et al. 2017; Malfanova et al. 2012; Ongena and Jacques 2008). Lipopeptides are group of non-ribosomally synthesized peptides (NRPs) produced by bacteria, yeast, mold, and actinomycetes (Laverty et al. 2011; Li et al. 2013; Zhao et al. 2018). In bacteria both Gram-positive and Gram-negative groups of bacteria have been reported for lipopeptides production (Li et al. 2013), among them *Bacillus spp* are dominant (Malfanova et al. 2012; Raaijmakers et al. 2010; Zhao et al. 2016).

Lipopeptides are amphipathic peptides with hydrophilic oligopeptide (with L and D amino acids) linked to hydrophobic fatty acid chain (Zhao et al. 2016). The amino acid sequence of oligopeptide categorizes lipopeptides into iturin, fengycin, and surfactin families (Ongena and Jacques 2008). Moreover, each family of lipopeptides produced by a particular strain is varied in accordance with cultural conditions (Rangarajan and Clarke 2015). Further, each family of lipopeptides comprises its variant with subtle amino acid substitution and isoforms due to varying lengths of fatty acid (Raaijmakers et al. 2010; Zhao et al. 2017). Surfactin families (heptapeptide) of lipopeptides comprise lichenysin, pumilacidin from *B. amyloliquefaciens, B. licheniformis*, and *B. pumilus* (Zhao et al. 2016). Iturin (heptapeptide) group of lipopeptides consists of bacilomycins and its variants, mycosubtilysin reported from *B. subtilis* and *B. amyloliquefaciens*, while fengycin group (decapeptide) such as pilipastatin from *B. cereus*, *B. thuringiensis* (Ongena and Jacques 2008; Zhao et al. 2016)*.* Another group of lipopeptides such as brevibacillin a linear cationic lipopeptide from *Brevibacillus laterosporus, B. brevis* reported previously (Yang et al. 2016).

Purification of each family of lipopeptide and its homologues has a great challenge, a single method of purification is insufficient to purify each family and its homologue (Varjani, and Upasani 2017). Further, its high cost of purification and low yield limits its commercialization and its various applications. However, therapeutic applications of lipopeptides need a high level of purity and it could be attained by coupled extraction and purification techniques (Rangarajan and Clarke 2015). The potency of diverse types of lipopeptides homologue against particular organisms is mainly influenced by its amino acid composition and length of fatty acid chain (Ongena and Jacques 2008; Dhanarajan et al. 2016). Among the class of lipopeptides, iturin is mainly reported for its antifungal activity against plant pathogens and fengycin for both antibacterial and antifungal activity, while surfactin shows diverse types of functions such as antibacterial, antifungal, antiviral, antitumor activity (Ongena and Jacques 2008). Although most of the literature reported lipopeptides from *B. velezensis spp.* for its antifungal activity, whereas antibacterial activities especially against human pathogen relatively scare in the literature (Grady et al. 2019; Li et al. 2020a, b; Li et al. 2020a, b; Palazzini et al. 2016; Ye et al. 2018).

It is thus necessary to search for the new antimicrobial agents, hence, the present study aims to screen potent antimicrobial peptides producing strain from soil. Lipopeptides produced by *B. velezensis* SK were extracted comparatively by various extraction methods and investigated for antimicrobial efficiency and purity. Further, several purification strategies were evaluated for the effective purification of lipopeptides from *B. velezensis* SK. Further, isolated lipopeptides were identified and characterized by using analytical techniques such as TLC, FTIR, and LC–ESI–MS along with their antimicrobial potential preferably against drug-resistant bacteria along with foodborne pathogens.

## Materials and method

### Indicator microorganisms

Indicator microorganisms used, Gram-positive organism: *B. cereus* NCIM 2703, *B. subtilis* NCIM 2635, *S. aureus* NCIM 2654 and Gram-negative: *E. coli* NCIM 2832, *P. aeruginosa* NCIM 5032, *Proteus vulgaris* NCIM 2813, *Salmonella typhimurium* NCIM 2501 were purchased from the National Collection of Industrial Microorganisms (NCIM), Pune and aminoglycoside resistant *B. cereus* ATCC 10876 from American type culture collection (Product code 0998P) Hi-Media, USA. Clinical isolate *S. aureus* and *E.coli* were also used for the antimicrobial activity.

### Screening of antimicrobial producing strain and growth media

Total of 15 rhizospheric soil samples was collected from the Western Ghats region especially surrounding areas of Radhanagari forest, Kolhapur, and screened for antagonistic activity against selected test organisms. Briefly, 1 g soil sample was homogenized in normal saline by vortex mixture, 100 µl aliquot from selected tenfold serial dilutions were spread plated on various media such as Nutrient agar (NA), tryptic soy agar (TSA), Muller Hinton Agar (MHA), Brain heart infusion agar (BHI), Potato dextrose agar (PDA) Hi-Media, Mumbai, India. These plates were then incubated at 25 °C and 37 °C temperature at 24 h and 48 h. Colonies with distinct morphology were selected and transferred on nutrient agar slants; the slants were preserved at 4 °C for further analysis. The antagonistic activity of each isolate was checked by method described previously (Guo et al. 2012), briefly the culture from each slant spotted on Muller Hinton Agar (MHA) plate spread with indicator organism in primary screening. After incubation at 25 °C and 37 °C temperature for 24 h and 48 h, plates were observed for the zone of inhibition around the growth of the spotted isolate. Further, isolate with potent antagonistic activity against indicator organism was checked by cross streaking method (Ganesan et al. 2017).

### Phenotypic, biochemical, and genotypic characterization of AMPs producer strain

Potent antimicrobial producing isolate (*B. velezensis* SK) was grown on Nutrient agar (NA), colony characters noted and morphology was examined by Gram straining. Commercial HiBacillus identification kit (Himedia, Mumbai) was used for biochemical characterization. In addition, biochemical tests were performed to confirm the identity of isolated strain and compared with related strains from literature. The sugar fermentation tests such as glucose, sucrose, fructose, lactose, galactose, Mannitol, xylose, and other tests like catalase, nitrate reduction, indol, methyl red, Voges-Proskauer, citrate utilization, and hydrolysis of starch and gelatine was performed as per previous study (Li et al. 2020a, b). Further, *B. velezensis* SK strain was also tested for salt tolerance and antibiotic sensitivity test.

Genotypic identification of *B. velezensis* SK was done by using 16S rRNA gene sequencing, genomic DNA was isolated from strain *B. velezensis* SK. ThermalCyclerPCRVeriti®96well was used for the amplification of 16S rRNA by using universal forward 8F primer and reverse 1492R primer, amplicon of 1500 bp purified by using 1.2% agarose gel electrophoresis and used for sequencing by Sanger method using BDTv3.1CyclesequencingkitonABI3730xl Genetic Analyser. The resultant rRNA sequence was then used to identify closely related organisms by NCBI BLASTn in GenBank database (https://blast.ncbi.nlm.nih.gov/) and EzTaxon Database (Chun et al. 2007), briefly 16S rRNA sequence extracted in Fasta format and used for the construction of phylogenetic tree by using the Neighbor-Joining method with the help of MEGA7 (Kumar et al. 2016). The 16 s rRNA sequence was submitted to GenBank (GenBank accession number MK007327.1) and isolated strain has been preserved at Microbial Type Culture Collection, Chandigarh, India (Accession No. MTCC 25,450).

### Production media, inoculum preparation, and culture conditions

Initially, the production of antimicrobial peptides from *B. velezensis* SK was evaluated in various production media such as nutrient broth (NB), tryptic soy broth (TSB) Brain heart infusion broth (BHI), and Czapek Dox using shake flask fermentation. The inoculum was prepared by inoculating a single colony of *B. velezensis* SK strain in 50 ml NB and incubated at 37 °C for 24 h. Erlenmeyer flask (250 ml) with 100 ml working volume of respective medium were used to inoculate 3% inoculum and incubated at 37 °C for 24 h. Production of AMP was also evaluated at different incubation times 24 h, 48 h, 72 h, and 96 h in nutrient broth. The AMPs extracted by Diaion HP-20 resin from cell-free supernatant as described further.

The media suggested by Das et al. with slight modification was used as a minimal basal medium (MBM) for the production of lipopeptides (Das et al. 2008). The MBM compose of; D-Glucose 20 g/l, NaNO_3_ 4 g/l, K_2_HPO_4_ 2.2 g/l, KH_2_PO_4_ 0.14 g/l, NaCl 0.01 g/l, MgSO_4_·0.6 g/l, CaCO_3_ 1.0 g/l, FeSO_4_ 0.02 g/l, MnSO_4_·4H_2_O 0.0017 g/l, ZnSO_4_·7H_2_O 0.0016 g/l (pH 7.2 ± 0.5). The 3% inoculum was used for the inoculation of 200 ml production media (MBM) and incubated at 37 °C for 48 h in a shaker incubator (REMI) at 120 rpm agitation speed. All fermentations experiments were carried out in a 500 ml Erlenmeyer baffled flask with a 200 ml working volume.

### Time course growth and lipopeptide production kinetics studies of *B. velezensis* SK

Growth kinetic studies of *B. velezensis* SK were performed by using MBM with composition and cultural conditions as described above; 2 ml samples were withdrawn at a regular interval of 1 h. This sample was then analyzed for O.D at 660 nm for growth, pH, biomass estimation, residual glucose, and production of the lipopeptide. Residual glucose concentration was estimated by DNSA method, briefly cell-free supernatant mixed with DNSA reagent and boiled for 10 min O.D measured at 530 nm, a calibration curve of glucose was prepared by using 500 µg/ml of glucose (Detns et al. 1959). The turbidometric method was used for the estimation of crude lipopeptide in the sample (Mukherjee et al. 2009), briefly sample was acidified to pH 2 using 6 M HCl and turbid sample vortex and O.D was measured at 600 nm by using UV–Vis spectrophotometer. Calibration curve of standard surfactin (Sigma 98% purity) was plotted against O.D at 600 nm vs. known concentration of surfactin in µg/ml.

### Comparison of extraction methods for efficient extraction of antimicrobial lipopeptide

Various methods were evaluated for the extraction of bioactive peptides from cell-free supernatant. Assuming the peptide nature of the antagonistic agent, initially, ammonium sulphate at 70% (w/v) saturation was used to precipitate all peptides and proteins, further bioactive agent was recovered by 30% (w/v) precipitation as reported for purification of lipopeptides (Liu et al. 2015). An organic solvent such as chloroform (CHCl_3_) and Ethyl acetate (EtoAc) in equal volume was used separately to extract an antimicrobial agent from cell-free broth. Briefly, solvent added broth was agitated for 2–3 h in a separating funnel and allow to separate phases (Varjani and Upasani 2017). Separated organic phase was evaporated at 40 °C and the dried residue was dissolved in methanol and checked for antagonistic activity against indicator organism. Acid precipitation using 6 M HCl was used for the partial purification of lipopeptides as reported earlier (Nanjundan et al. 2019; Varjani and Upasani 2017). Briefly cell-free supernatant acidified to final pH 2 and kept for overnight at 4 °C, precipitate was collected by centrifugation at 10,000×*g* at 4 °C for 15 min and the resultant pellet was neutralized and extracted with methanol, evaporated, and redissolved in 70% methanol (v/v) to obtain crude yellowish lipopeptide.

### Partial purification of lipopeptides by macroporous adsorption resin

The method of affinity extraction using Macroporous adsorption resin (MARs) was used to isolate bioactive agents from cell-free supernatant (Dhanarajan et al. 2015), MARs such as Amberlite XAD-16 and Diaion HP-20 resins were purchased from Sigma Aldrich with physical properties as listed in Table S1, resin-treated according to manufacturer instructions. Prior to use resin was soaked in absolute methanol and then washed with water and dried at 40 °C. The 2% resin was added in cell-free supernatant and kept at 37 °C for 3 h for the adsorption. Further, Diaion HP-20 adsorption method was optimized for the extraction of bioactive compounds, after adsorption resin was removed and filled in chromatographic column, and washed with 500 ml distilled water. The extraction of lipopeptides was carried out from resins by using increasing gradients of methanol as 20%, 40%, 60%, 80%, and finally 100% (v/v) methanol, extracted lipopeptide fractions were assayed for antibacterial activity by well diffusion assay as described earlier.

## Purification of lipopeptide by chromatographic techniques

### Purification of lipopeptide by silica gel chromatography

The partially purified extract was loaded on silica gel (60–120 mesh) column and eluted stepwise with a linear gradient of chloroform: methanol solvent system in a ratio (CHCl_3_:CH_3_OH) as follows 9:1, 8:2, 7:3, 6:4, 5:5, 4:6, 3:7, 2:8, 1:9 (v/v) and finally with 100% methanol (Sharma et al. 2014). Total 45 fractions of 2 ml were collected with a constant flow rate of 1 ml/min and UV absorbance of all fractions was recorded at 280 nm. Another elution program was also utilized using the increasing gradient of methanol: water from 3:7, 4:6, 5:5, 6:4, 7:3, 8:2, 9:1 v/v, and finally with absolute methanol (Korenblum et al. 2012), a total of 16 fractions of 5 ml were collected. Fractions evaporated at 40 °C, dissolved in methanol, and assayed for antimicrobial activity by paper disc (30 µl) or agar well diffusion assay (100 µl). Fractions showing antimicrobial activity pooled and analyzed by RP-HPLC.

### Purification of lipopeptide by Sephadex LH-20 chromatography

Diaion HP-20 extracted lipopeptide was further purified by using Sephadex LH-20 (GE Healthcare) column (dimensions 15 × 1.9 CM), prepared by Sephadex LH-20 matrix soaked in methanol overnight (Li et al. 2020a, b). The LH-20 column was equilibrated with methanol and washed several times with the same solvent. Then HP-20 bioactive extract containing lipopeptide in methanol was loaded on the column and eluted with HPLC grade methanol at a constant flow rate of 1 ml/min. Total 28 fractions of 2 ml collected, purity of separated fractions checked by TLC with solvent system ethanol: water (7:3). Spot visualized by exposing the plate to UV and also by iodine vapour. The antimicrobial activity of all fractions was checked by the paper disc method.

### Purification of lipopeptide by Diaion HP-20 chromatography using dual gradient technique:

In order to separate either of the lipopeptides families produced by *B. velezensis* SK such as iturin, fengycin, and surfactin, a dual gradient of pH and solvent was used in this study as described by Dhanarajan et al. (2015) with slight modifications (Table S2). A glass column (2.0 × 50 cm) was packed with pre-activated Diaion HP-20 resin (15 g). Then partially purified lipopeptide (5 gm) dissolved in 5 ml distilled water was loaded on the top of the column, the column was washed with 250 ml water and eluted stepwise by a dual gradient of pH and solvent acetone with 1 ml/min flow rate (Table S2). Collected fractions were neutralized, evaporated, and dissolved in methanol and analyzed by using TLC and oil displacement test as described previously. Antimicrobial activity of all fractions was performed by agar well diffusion assay, further active fractions were analyzed by RP-HPLC, FTIR, and LC–ESI–MS.

### Reverse-phase high-performance liquid chromatography (RP-HPLC) analysis of lipopeptides

Purification and analysis of lipopeptide homologues were carried out by using a reverse-phase high-performance liquid chromatography (RP-HPLC) system (JASCO) equipped with a quaternary pump, autosampler, and UV detector. A semi-preparative scale Hiber C18 column (250 × 4.6 mm, 5 μm) was used for lipopeptide purification as described earlier with modification (Chen et al. 2010; Sarwar et al. 2018). RP-HPLC was run in isocratic mode, 20 µl Diaion HP-20 extracted sample of lipopeptide injected into column, purification of lipopeptide attempted by using mobile phase methanol/water/trifluoroacetic in a ratio of 80:20:0.1 (v/v). The flow rate was maintained at 1 ml/ml, lipopeptide peak was detected by UV detector at 210 nm at semi-preparative scale. The peaks correspond to standard surfactin (Sigma 98% purity) collected, pooled, and re-injected for purity, concentrated by evaporation at 40 °C, and used for further analysis.

## Chemical characterization of lipopeptide produced by *Bacillus velezensis* SK

### Thin layer chromatography (TLC) analysis of lipopeptides

Lipopeptides extracted and purified by various methods in the present study was further separated and identified by TLC by using two different solvent systems, Solvent System- I; Butanol: acetic acid: water (4:3:2) for amino acid and peptides (Seghal Kiran et al. 2010), Solvent System-II; Ethanol: water (7:3) for peptides (Balan et al. 2017). Briefly extracted lipopeptide from various preparation was spotted on silica gel plate GF 254 (Merk) and then the plate was developed by either of a solvent system described above. Then plates were visualized by various ways for the identification of nature of separated bioactive agents, such as by 0.2% ninhydrin in acetone followed by heating at 110 °C for peptides, or visualized at UV wavelength 254 nm or sprayed with water for lipophilic nature. Further, the lipophilic nature of the peptide was confirmed by exposing the TLC plate with iodine vapour.

### Bioautography of TLC separated lipopeptides

Bioautography analysis was performed as described by Dewanjee et al. (Dewanjee et al. 2015). TLC plate was developed with solvent system-II, and dried in the oven at 40 °C for 30 min, sterilized by exposing to UV light in laminar. The sterile TLC plate was then aseptically transferred to a sterile Petri plate and overlaid with 20 ml soft agar (Ca-MHA) seeded with *B. cereus* NCIM 2703 and then allowed to solidify. The plate was incubated at 37 °C for 24 h after incubation plate was observed for the spot showing a zone of inhibition. A value of the retardation factor (Rf) of the active spot was determined.

Preparative scale TLC was run in duplicate in the same condition as above, corresponding spot showing antimicrobial activity was scraped from TLC plate and eluted with 70% methanol and subjected to antimicrobial activity by disc and well diffusion assay against indicator organism at 37 °C for 24 h following the protocol of Clinical Laboratory Standard Institute (CLSI, 2019). After incubation, plates were observed for the zone of inhibition.

### Solubility of lipopeptide produced by *B. velezensis* SK

Solubility of purified lipopeptide was evaluated in different solvent chloroform, ethyl acetate, acetone, methanol, ethanol, and water.

### Effect of pH and Temperature on antimicrobial activity of lipopeptides

In order to check the temperature stability of isolated surfactin lipopeptide with respect to its antimicrobial activity, 200 µl of lipopeptide sample was exposed to a range of temperature 0 °C, 20 °C, 37 °C, 50 °C, 60 °C, 70 °C, and 80 °C for 2 h. Further, for the pH stability, lipopeptide was dissolved in citrate phosphate buffer of pH 2, 4, 6, 7 and Glycine–NaOH buffer of pH 8 and 10 after 2 h incubation at respective buffer, mixture was then neutralized and checked for its antimicrobial activity by well diffusion assay against selected indicator microorganisms.

### Oil displacement test

The biosurfactant-like character of lipopeptide produced by *B. velezensis* SK was examined by an oil displacement test as described earlier (Nanjundan et al. 2019).

### Fourier-transform infrared spectroscopy (FTIR) analysis of lipopeptides produced by *B. velezensis* SK

Fourier transform infrared spectroscopy was used to determine the chemical nature of bioactive agent viz. lipopeptide produced by *B. velezensis* SK, by identification of the functional group. FTIR spectra of lipopeptide extracted and purified by various methods were recorded and compared with standard surfactin. Briefly, samples were mixed with vacuum-dried KBr and pressed into a pellet. FTIR spectra of samples were recorded in the range 4000 to 500 cm-1 by BRUKER Alpha 100,508 FT-IR model Germany.

### Identification and molecular mass determination of lipopeptide by liquid chromatography mass spectroscopy LC–ESI–MS

Active fractions from Diaion HP-20 chromatography and HPLC purified lipopeptide was first separated by capillary liquid chromatography and then analyzed by a mass spectrometer (Agilent model G6540B) equipped with ESI/nanospray ionization quadrupole time of flight (Q-TOF) operated in positive ion mode as described earlier with minor modification (Yang et al. 2016). A sample (8 µl) was dissolved in methanol and injected in capillary column (Agilent Zorbax C18 2.1 × 50 mm 1.8 µm particle size), then separated by using mobile phase A 0.1% trifluoroacetic acid in water and mobile phase B was 0.1% trifluoroacetic acid in 90% acetonitrile (ACN) at flow rate 2 µl/min. Typically, mobile phase B increased from 2 to 40% in 60 min and from 40 to 90% in the next 2 min and then kept at 97% for the next 3 min before being decreased quickly to 3%. Then, Column was equilibrated with 3% of mobile phase B before being the next injection.

Electrospray ionization (ESI–MS) mass spectroscopy analysis of separated peaks was performed by mass spectrometer system Agilent model G6540B) equipped with quadrupole time of flight (Q-TOF). The ESI–MS spectra were acquired in a positive ion mode with condition 3 kV capillary voltage, 8 l/min dry gas, and 250 °C dry gas temperature. The analysis of MS spectra was programmed in a full scan of injected sample and recorded on positive ion mode within the mass range 180 to 1700 m/z.

### Determination of antimicrobial activity

Antimicrobial activity of lipopeptide extracted by various methods described above was determined by various antimicrobial bioassay methods in accordance with CLSI standards (CLSI 2019; Balouiri et al. 2016); prior to use all extract was filtered sterilized by 0.22 µm syringe filter (Axiva) collected in a sterile vial.

Agar well diffusion assay was mainly used for the determination of antagonistic activity during purification against selected indicator microorganisms as described in an earlier report (Balouiri et al. 2016). For this, a suspension of indicator microorganisms was prepared by adjusting turbidity to 0.5 McFarland standard. An aliquot of 100 µl suspension was spread on calcium adjusted Ca-MHA plate, 6 mm well was prepared and filled with 100 µl antimicrobial extract (3 mg/ml). Then plates were incubated at 37 °C for 24 h, and observed for the zone of inhibition in diameter (mm) around the well and measured.

The spot on lawn bioassay method was used for bioassay for the determination of arbitrary unit AU of peptide (Guo et al. 2012). Soft agar of Ca-MHA, seeded with indicator organism (100 µl) overplayed onto in sterile Petri plates, and then twofold of serially diluted purified peptide (3 mg/ml) was spotted on it. After incubation at 37 °C for 24 h plates were observed for the zone of inhibition in diameter (> 2 mm) and AU/ml calculated as follows. Antimicrobial activity of surfactin lipopeptide is expressed in arbitrary unit AU/ml; defined as reciprocal of highest dilution shows the zone of inhibition.

$$\mathrm{AU}/\mathrm{ml }=\frac{\mathrm{Reciprocal of highest dilution}*1000 }{\mathrm{Amount of diluent spotted}}$$

### Statistical analysis

All experiments were performed in three or more replicates and results were expressed in mean ± SD (standard deviation). Statistical significance of data was determined by applying a two-tail t-test in data analysis tool of Microsoft Excel 2013 and expressed as p-value, p < 0.05 considered as statistical significant.

## Results

### Screening and isolation of antimicrobial producing microorganisms

**Fig. 1:**
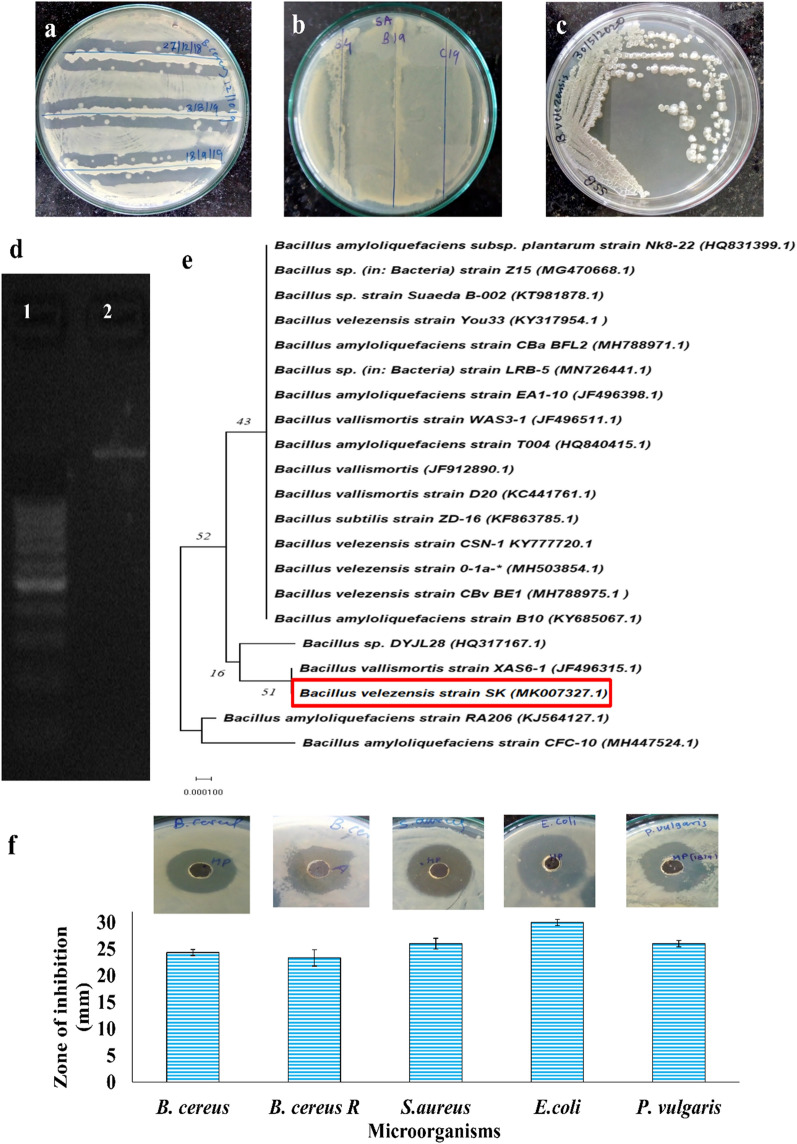
Antagonistic activity of *B. velezensis* SK by cross streak method against **a** *B. cereus* NCIM 2703 **b** *Staphylococcus aureus* NCIM 2654, and **c** Colony morphology on nutrient agar **d** 1500 bp amplified 16 s rDNA. **e** Evolutionary relationship of *B. velezensis* SK based on 16 s rRNA and closely related species of *Bacillus*, phylogenetic tree was constructed by using Neighbour-Joining method by using MEGA7 software, bootstrap values on each branch point indicates 1000 pseudo replicates (scale represents 0.0001 nucleotide substitution). **f** Antimicrobial activity of partial purified (HP-20) lipopeptides by using agar well diffusion assay showing zone of inhibition (upper panel) in diameter in mm (lower panel) against Gram-positive and Gram-negative indicator bacteria including streptomycin resistant (R)

In primary screening, 150 bacterial isolates with distinct morphology were isolated as pure culture from soil samples and screened for antagonistic activity against indicator organisms (Additional file 1: Table S3). The results showed that 17 isolates out of 150 isolates have depicted antagonistic activity against indicator organisms. Moreover, isolate B4 showed broad-spectrum antimicrobial activity against seven indicator organisms including both Gram-positive as well as Gram-negative (Additional file 1: Table S3). Further, strain (B4) was identified as *B. velezensis* SK, the strain showed broad-spectrum antimicrobial activity specifically against *B. cereus* NCIM 2703 and *S. aureus* NCIM 2654 (Fig. 1a, b), which was further confirmed by agar well diffusion bioassay.

### Phenotypic, biochemical, and genotypic characterization of lipopeptide producer strain

Phenotypic, morphological, and biochemical characterization was studied for selected isolate and identified as a new strain designed as *B. velezensis* SK. A new strain *B. velezensis* SK forms 2–4 mm irregular and creamy white colonies on nutrient agar (Fig. 1c, Table 1). Microscopic examination of isolate shows actively motile aerobic bacteria and by using Gram stain shows Gram-positive, rod-shaped bacteria (Table 1). Biochemical characterization of strain showed positive tests for catalase, nitrate reduction, delayed positive Voges-Proskauer, and hydrolyzed starch and gelatine (Table 1). It shows a negative reaction for indol, methyl red, glucuronidase, citrate utilization, and ONPG. The isolated strain can tolerate an 8% NaCl salt concentration. The strain utilized various sugar which was demonstrated by the production of acid from D-Glucose, D-fructose, D-galactose, D-mannitol, sucrose, and negative for D-lactose. These results of carbohydrate fermentation showed that isolated strain possesses similarity with *B. amyloliquefaciens* operational group (Li et al. 2020a, b; Rabbee et al. 2019; Ruiz-García et al. 2005; Wang et al. 2020). However, the lactose negative, ONPG negative, with nitrate reduction positive make the *B. velezensis* SK strain distinct from related species as reported earlier (Additional file 1: Table S4). In addition to biochemical tests, strain *B. velezensis* SK showed methicillin and penicillin G resistant along with moderate resistant to fusidic acid. Whereas, the strain is susceptible to streptomycin, chloramphenicol, tetracycline, erythromycin, and Novobiocin (Additional file 1: Figure S1a, Table S5).

**Table 1:** Morphological, physiological, and biochemical characteristics of the *B. velezensis* SK

Microscopic examination	
Gram’s Nature	Gram-Positive
Morphology	Rods
Arrangements	Chains
Motility	Actively motile
Colony characteristics	
Size	2–4 mm
Shape	Circular
Surface	Rough
Margin	Irregular
Elevation	Raised
Colour	White
Opacity	Opaque
Biochemical Characterization	
Indol	− Ve
Methyl red	− Ve
Voges-Proskauer	Delayed positive
Citrate utilization	− Ve
Catalase	+ Ve
Glucoronidase	− Ve
Nitrate reduction	+ Ve
ONPG	− Ve
Sugar fermentation	
Glucose	+ Ve
Sucrose	+ Ve
Fructose	+ Ve
Mannitol	+ Ve
Galactose	+ Ve
Lactose	− Ve
Xylose	− Ve
Starch hydrolysis	+ Ve
Gelatine hydrolysis	+ Ve
NaCl tolerance	< 8%

Neighbor-joining phylogeny tree revealed the close relationship of *B. velezensis* SK with *B. amyloliquefaciens* operational group, with highest degree of similarity with the strains of *B. velezensis* 0-1a, *B. vallismortis* XAS6-1, *B. vallismortis* D20, *B. vallismortis* WAS 3–1, *B. subtilis* ZD-16*,* and *B. amyloliquefaciens* EA1-10. A phylogenetic tree of 16S rRNA (1458 bp) sequence of *B. velezensis* SK and its closely related species from NCBI BLASTn was constructed (Fig. 1d, e). The 16S rRNA (1458 bp) sequence of *B. velezensis* SK was also analyzed and compared with strains existing in EzTaxon Database (Chun et al., 2007). Isolated strain *B. velezensis* SK forms a single clade together with closely related *B. amyloliquefaciens*, *B. vallismortis*, *B. velezensis*, and *B. subtilis* strain (Fig. 1e). Thus, the isolate was identified as a new bacterial strain *B. velezensis* SK based on the phenotypic, biochemical, and phylogenetic analysis.

### Evaluation of lipopeptide production in various media by *B. velezensis* SK

The new strain *B. velezensis* SK showed constitutive production of lipopeptides in nutrient broth, which can be visually monitored for production of stable foam (Additional file 1: Figure S1b). Partially purified lipopeptides by HP-20 Diaion resin, showed potent and broad-spectrum antimicrobial activity against Gram-positive and Gram-negative indicator organisms with average ≥ 24 mm diameter of zone of inhibition in agar well diffusion assay (Fig. 1f). Antimicrobial production from *B. velezensis* SK was observed after 24 h, 48 h, and 96 h of incubation times in NB without significant difference (P-value > 0.05) in their antimicrobial activity, suggesting 24 h incubation sufficient for production in NB (Additional file 1: Figure S2a).

The stain *B. velezensis* SK is capable of the production of lipopeptide in all NB, BHI, TSB, and semisynthetic Czapek Dox medium with maximum production in NB (P-value < 0.05) as shown in this study (Additional file 1: Figure S2b).

### Time course growth and lipopeptide production kinetics studies of *B. velezensis* SK

**Fig. 2:**
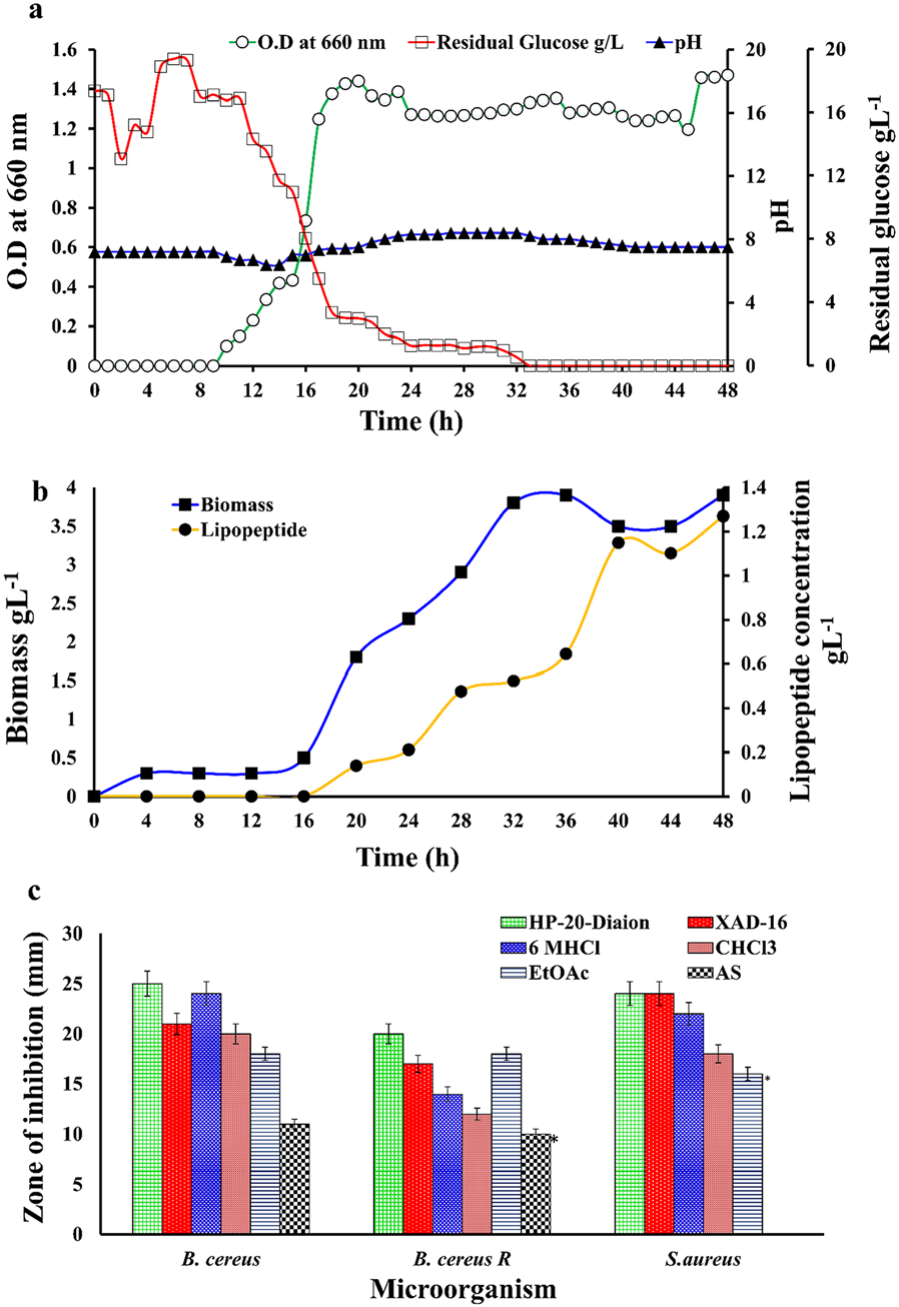
**a** Time dependent growth curve analysis of *B. velezensis* SK, for growth (O.D at 660 nm), residual glucose concentration and pH. **b** Lipopeptides production and biomass production kinetics in MBM at 37 °C, 120 rpm agitation for 48 h. **c** Comparative antimicrobial activity of lipopeptides extracted by various methods from *B.velezensis* SK for efficient extraction against Gram-positive bacteria

Growth profile of *B. velezensis* SK and its lipopeptide production kinetics against the function of time was studied in minimal basal medium (MBM) with glucose as the sole carbon source. Kim et al. (1997) reported that glucose is a superior carbon source for the lipopeptide production by *B. subtilis* C9. The strain *B. velezensis* SK shows luxuriant growth in MBM after 9 h of lag phase and lipopeptide production started at mid log phase at 16 h and reach a maximum at the end of stationary phase at 36 h (Fig. 2a). Earlier studies showed surfactin production mainly at the late exponential phase (Ongena and Jacques 2008). Production of the lipopeptide is positively correlated with the biomass of *B. velezensis* SK*,* while residual glucose concentration shows a negative correlation with growth which can completely exhausted at the stationary phase at 32 h (Fig. 2a). One of the earlier studies with *B. subtilis* DSM 10 showed limiting glucose in the medium resulted in high surfactin yield, this could be due to nutritionally deprived conditions favours the lipopeptide production (Willenbacher et al. 2015).

The drop in initial pH value 6.4 was observed at log phase (14 h), which might be due to glucose utilization and acid production (Fig. 2a). While a gradual rise in pH value from 6.4 at 16 h to 8.4 at stationary phase at 32 h indicates the organism might be shifted towards a new carbon source, which also indicates the progressive accumulation of lipopeptide over the 16 h to 32 h fermentation. The previous study with *B. altitudinis* MS16 also reported slight alkaline pH favour the lipopeptide production (Goswami and Deka 2015; Liu et al. 2019). Most of the earlier studies reported that glucose concentration at 20–40 gL^−1^ favours lipopeptide production and was found as a best carbon ‘C’ source, while excess glucose concentration leads to the production of undesirable metabolites (Hmidet et al. 2017; Rangarajan and Clarke 2015). Growth and lipopeptide production studies of *B. velezensis* SK revealed that this strain can efficiently use 2% glucose and produce biomass, cell dry weight (CDW) 3.72 ± 0.20 gL^−1^, and produces 1.33 ± 0.09 gL^−1^ of lipopeptide (Fig. 2b). An earlier study by Zheng et al. (2012) has shown that the production of 1.22 gL^−1^ surfactin from *B. subtilis* ATCC 21,332 in MBM media with similar composition. Our one of the experiments showed that the replacement of nitrogen source from sodium nitrate (NaNO_3_) to dual nitrogen source ammonium nitrate (NH_4_NO_3_) does not affect antimicrobial activity with reference to lipopeptide production. Growth curve of *B. velezensis* SK was also analyzed in nutrient broth which shows a shorter lag phase (4 h) as compared to lag phase in MBM (Additional file 1: Figure S3).

### Comparison of extraction methods for efficient extraction of antimicrobial lipopeptide

Various methods such as ammonium sulphate, acid precipitation, solvent extraction, and various reins extraction methods were used for the extraction of antimicrobial lipopeptides, the diverse physiochemical properties of lipopeptides makes the purification process challenging (Dhanarajan et al. 2016; Goswami and Deka 2019; Lee et al. 2008; Rangarajan and Clarke 2016; Smyth et al. 2014). The results of lipopeptides precipitated by 30% w/v ammonium sulphate showed less antimicrobial activity against both resistant *B. cereus* ATCC 10,876 and *B. cereus* NCIM 2703 (Fig. 2c) as compared to antimicrobial peptide extracted by other methods in this study (Lee et al. 2008). Results of solvent such as ethyl acetate (EtoAc) and chloroform (CHCl_3_) extracted lipopeptides showed no significant difference in their antimicrobial activity against the test organism, hence it is concluded that both solvents could able to extract antimicrobial peptides from broth (Fig. 2c).

However, due to the requirement of excessive solvent for extraction and limited solubility of solvent extracted lipopeptides (Rangarajan and Clarke 2016), Diaion HP-20 and XAD-16 were further used for extraction of antimicrobial lipopeptides from broth, results of both showed the significant difference in antimicrobial activity (≥ 20 mm diameter zone of inhibition) against *B. cereus* NCIM 2703, streptomycin-resistant *B. cereus* ATCC 10,876 and *S. aureus* NCIM 2654 as compared to other methods (Fig. 2c). These results conclude that HP-20 resin with high adsorption of lipopeptides has been found better than XAD-16, which is due to the difference in their physiochemical properties (Additional file 1: Table S1) (Rangarajan and Clarke 2017). Altogether, significant differences in the antimicrobial activity of lipopeptides extracted by various methods were observed (P-value < 0.05), and it was found that Diaion HP-20 resin is excellent for lipopeptide extraction as compared to others in this study (Fig. 2c). Hence, the Diaion HP-20 resin method was further optimized for effective purification by increasing the gradient of methanol, and results showed antibacterial lipopeptides efficiently extracted over 40% to 100% methanol as had antimicrobial activity (Additional file 1: Figure S4). All these results suggest that the lipopeptide nature of the antibacterial compound by *B. velezensis* SK which was confirmed by TLC and bioautography as described further.

Acid precipitation method for the extraction of lipopeptides from the cell-free broth was also used as per the previous report (Das et al. 2008; Ramachandran et al. 2017; Romano et al. 2013). Results showed that yellow methanolic extract of acid precipitated lipopeptides exhibit strong antimicrobial activity against *B. cereus* NCIM 2703, *S. aureus* NCIM 2654, and *B. cereus* ATCC 10,876 with 24 mm, 22 mm, and 14 mm zone of inhibition respectively with no significant difference (P-value > 0.05) with that of resin extracted lipopeptides (Fig. 2c). Hence, acid precipitation or resin extraction could be alternatively used for lipopeptide extraction. However, an optimized Diaion HP-20 resin method was used due to its easy operation, highest lipopeptide recovery, and purity for the lipopeptide extraction. Thus, it is clear that the combination of two or more methods could be used for the efficient purification of the antimicrobial lipopeptide.

## Chromatographic purification of lipopeptides produced by *B. velezensis* SK

### Purification of lipopeptides by silica gel column chromatography

Diaion HP-20 resin extracted lipopeptide was further purified by adsorption chromatography using silica gel (60–120 mesh) and step-wise elution by a linear gradient of chloroform and methanol CHCl_3_:CH_3_OH, as described in the materials and methods section. The initial 19 fractions showed antimicrobial activity against *B. cereus* NCIM 2703 by paper disc method, which also shows maximum absorbance at 280 nm in UV spectroscopy highlighting the elution of lipopeptides by CHCl_3_ among 40 fractions (Additional file 1: Figure S5a). All of the silica column bioactive fractions were pooled together and chromatographed by TLC with ethanol: water (7:3) solvent system. The pooled bioactive fractions lack other peptide impurities as they showed the negative reaction with the ninhydrin and showed an intense yellow spot with iodine vapour indicating antimicrobial activity mainly due to lipopeptides.

Silica gel column chromatography was also performed by a linear gradient of methanol from 30 to 100% v/v in water based on our HP-20 extraction optimization experiment. Total 16 fractions assayed for antimicrobial activity against *B. cereus* NCIM 2703, fractions corresponding to 60% (F8) and 70% methanol (F9, F10) showed the highest antimicrobial activity against *B. cereus* NCIM 2703 (Additional file 1: Figure S5b). These results conclude bioactive agents were eluted better at 60–70% methanol, which agreement with our HP-20 optimization experiment, corresponding fractions were pooled and monitored for purity by TLC. The TLC results showed that single yellow spot with retardation factor (Rf) value 0.88 with iodine vapour and UV without any impurities and free amino acids with 0.2% ninhydrin analysis.

### Purification of lipopeptides by gel filtration column chromatography

Fractionation of Diaion HP-20 extracted lipopeptide was also carried out by Sephadex LH-20 column with methanol as mobile phase. Total five fractions were assessed for the antimicrobial activity out of which fraction no.2 showed maximum antimicrobial activity against *B. cereus* NCIM 2703 with 24 mm zone of inhibition as compared to standard surfactin (60 µg) which showed an 11 mm zone of inhibition (Additional file 1: Figure S6).

### Purification of lipopeptide by Diaion HP-20 chromatography using dual gradient techniques

**Fig. 3:**
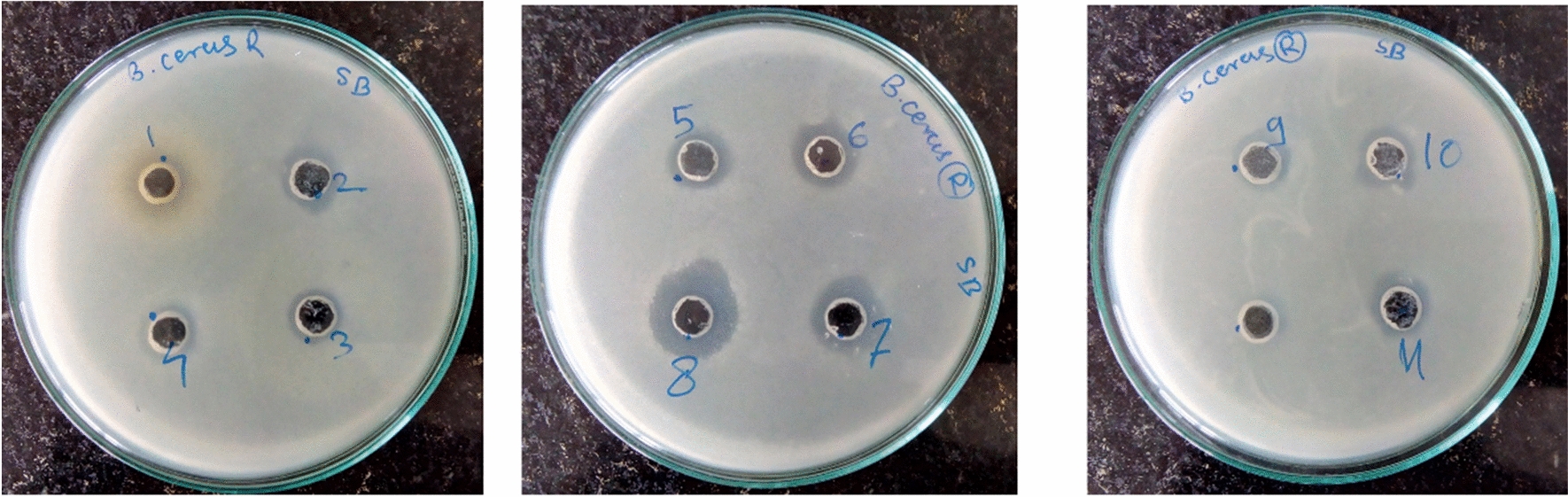
Antimicrobial activity of dual gradient (pH and acetone) fractions (1–11) by agar well diffusion assay against *B. cereus* ATCC 10,876 (6 mm well)

Dual gradient of acetone and pH were used to fractionate Diaion HP-20 extract as described in Additional file 1: Table S2, for the separation of families of lipopeptides (Dhanarajan et al. 2015). Antimicrobial activity of all fractions (two subfractions of F2-F6 each) from HP-20 dual gradient chromatography were checked by well diffusion assay. The results showed that fraction 2 (subfractions 2, 3) and fraction 3 (40% acetone at pH4) showed lesser antimicrobial activity, which may be due to the co-elution of surfactin along with iturin (Additional file 1: Table S2, Fig. 3). Surfactin rather than iturin was mainly reported for the antibacterial activity. Decreasing polarity of mobile phase with increasing acetone content along with alteration of pH from 4 to 8 favours separation of iturin in initial fraction 2 (F2) from surfactin in later fractions F3, F4, F5, and F6. Although all fractions show antibacterial activity against *B. cereus* ATCC 10,876 this might be due to the presence of a small amount of surfactin. Further, strong antimicrobial activity was observed with fraction 5 (80% acetone with pH 8) which is mainly composed of surfactin (Fig. 3). Further, the presence of iturin in fraction no. 2 and surfactin in fraction no. 5 was analyzed by RP-HPLC and finally confirmed by ESI–MS for its identification as discussed further. Hence, we hypothesized that *B. velezensis* SK produces mainly surfactin lipopeptide and a very small quantity of iturin, this might be because of cultural conditions during fermentation.

Further, all fractions checked by FTIR, oil displacement test, and TLC by iodine, the results showed the fraction F2 exhibit smooth curve similar to crude (Additional file 1: Figure S7). However, fractions F3 and F5 (Additional file 1: Table S2) show characteristic sharp peaks, which is similar to standard surfactin in FTIR analysis suggest purity of isolated surfactin.

### Reverse phase-High performance liquid chromatography (RP-HPLC) Purification, analysis of lipopeptides

The lipopeptides extracted by various methods and purified by chromatographic techniques exhibiting antimicrobial activity were further subjected to RP-HPLC analysis and compare with standard surfactin.

**Fig. 4:**
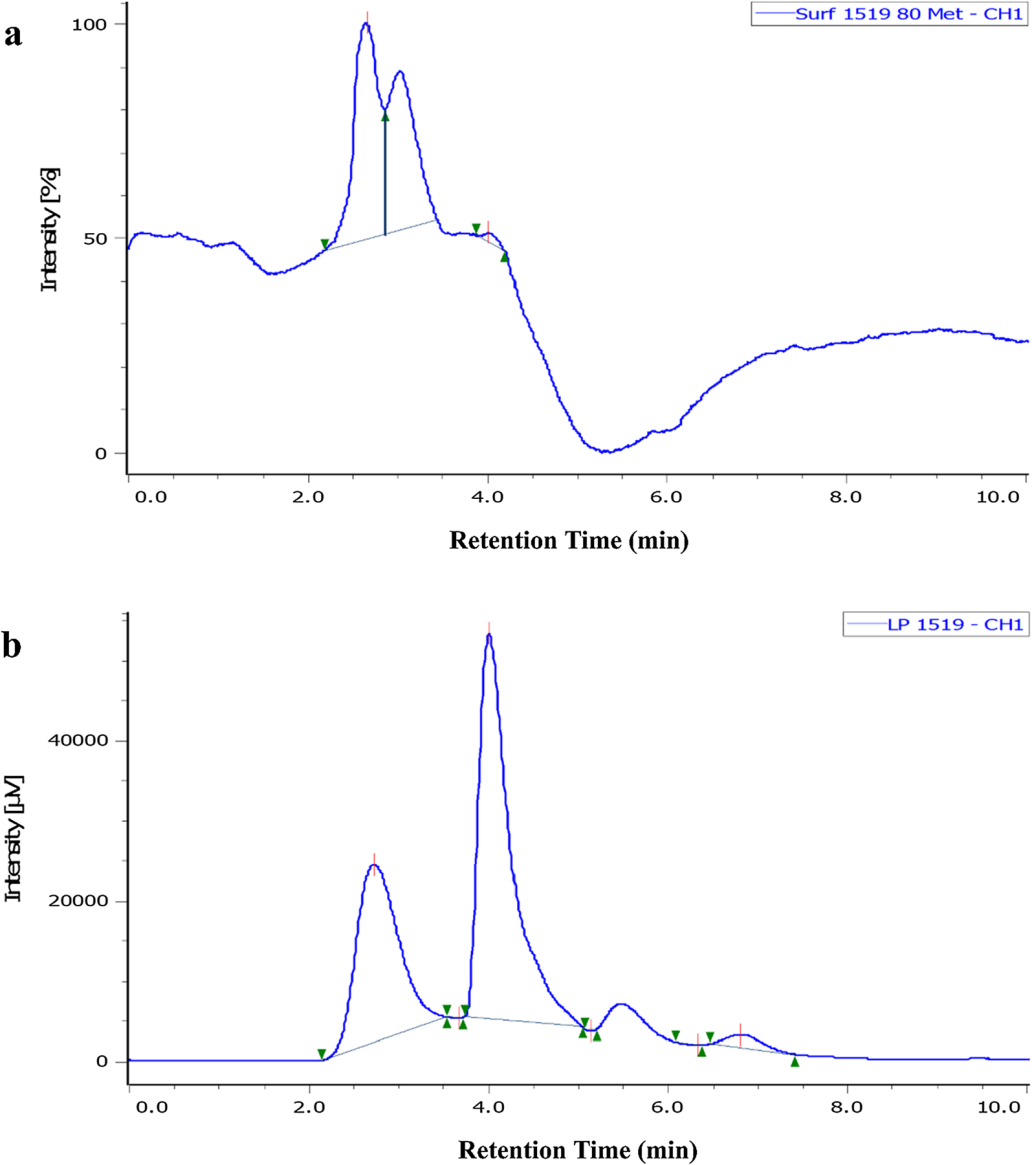
Reverse-phase HPLC purification of lipopeptides produced by *B. velezensis* SK Chromatogram of **a** Standard surfactin, **b** Chloroform extracted lipopeptides using mobile phase 80% methanol in water over 30 min

Chromatogram of chloroform/methanol extract showed four distinct peaks at a retention time (Rt) of 2.91 min, 4.02 min, 5.47 min, and 6.8 min which corresponds to standard surfactin with its homologues at the retention time of 2.61 min, 3.0 min, and 4.20 min (Fig. 4a and b). Additional peaks of chloroform extracted lipopeptides as compared to standard surfactin signified the presence of a newer surfactin isoform (Fig. 4b). Similarly, RP-HPLC analysis of Diaion HP-20 extracted lipopeptide showed two peaks at the retention time of 2.65 min (major) and 3.98 min (Additional file 1: Figure S8). A similar type of peaks has been observed for lipopeptide extracted by ethyl acetate, XAD-16 and purified by silica gel and Sephadex LH-20 column, which showed a major peak at Rt of 2.83 min, 2.74 min, 2.91 min, and 2.69 min respectively with other minor peaks (Additional file 1: Figure S8). The slight deviation in retention time of lipopeptides peaks from various extracts might be due to the presence of surfactin isoforms. These observations and peak analysis of extracted and column purified lipopeptides by RP-HPLC highlights the abundance of surfactin and its homologues when compare to standard surfactin which revealed that *B. velezensis* SK produces surfactin and its homologues. The coupled LH-20 and RP-HPLC purified surfactin lipopeptide showed antimicrobial activity with 13 mm zone of inhibition against *B. cereus* NCIM 2703 by well diffusion assay (Additional file 1: Figure S8e, inset), Iturin produced by *B. velezensis* SK could not be able to detect by RP-HPLC due to its less amount as compared to surfactin and its homologues.

## Physiochemical properties and chemical characterization of lipopeptide produced by *B. velezensis* SK

### Thin layer chromatography of lipopeptides

**Fig. 5:**
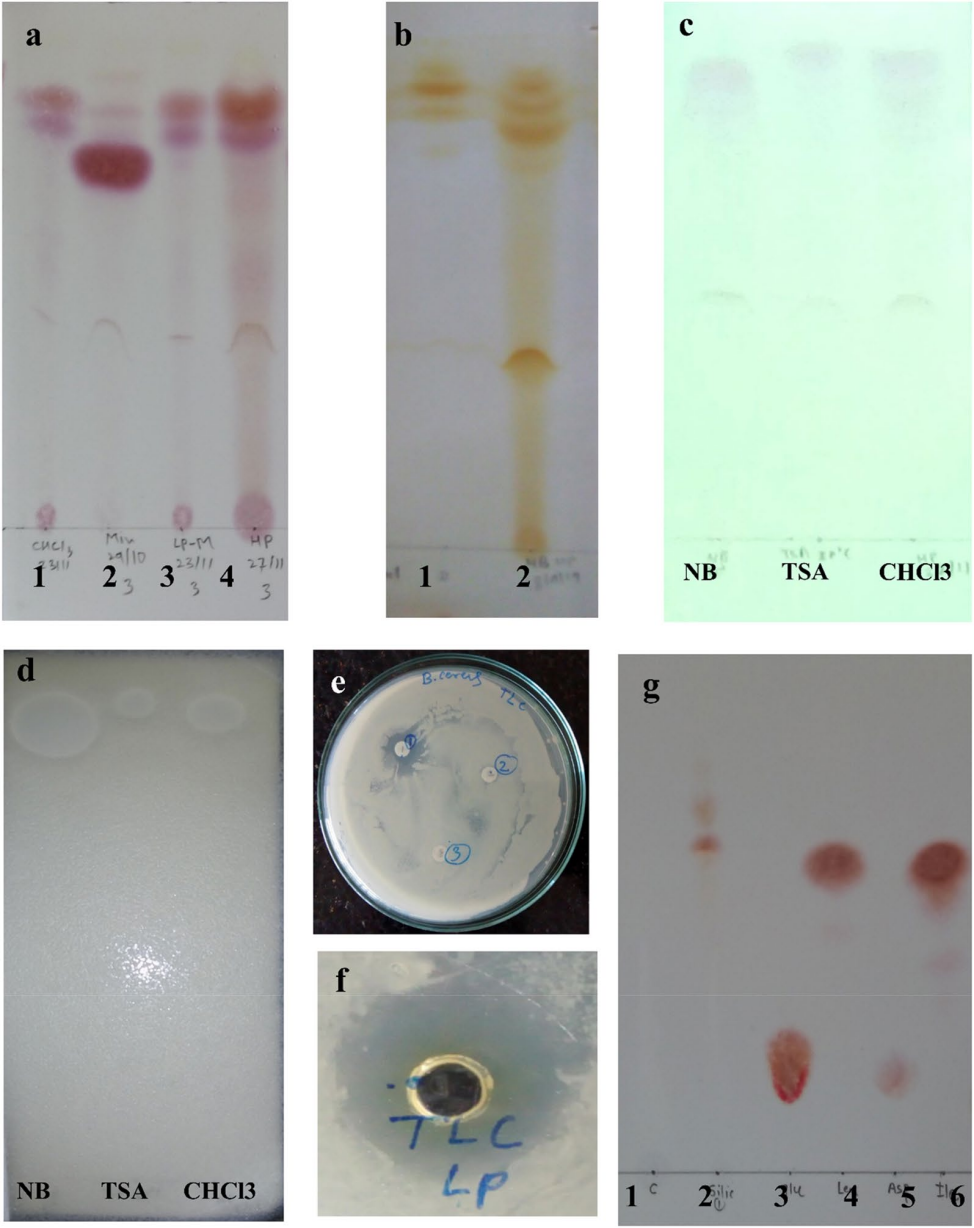
Thin layer chromatography (TLC) of partial purified lipopeptides from *B. velezensis* SK using **a** Solvent system-I and visualized with 0.2% ninhydrin, Lane 1, 2, 3, and 4 correspond to lipopeptide extracted with chloroform, lipopeptides produced in MBM, acid precipitated and methanol extracted lipopeptides and Diaion HP-20 lipopeptides respectively. **b** Solvent System-II and visualized by iodine vapour, TLC analysis of lipopeptide extracted by HP-20 lane 1 (MBM) and lane 2 (NB). **c** TLC plates of Diaion HP-20 extracted lipopeptide from NB visualized by UV 254 nm lane1, TSA lane 2, and Chloroform extracted lipopeptides lane 3 using solvent system-II. **d** In-situ antimicrobial activity of separated lipopeptides against *B. cereus* NCIM 2703 by using autobiography technique, and **e** Antimicrobial activity of TLC separated (upper spot) and eluted spots (disk 1) against *B. cereus* NCIM 2703 by paper disk assay. **f** Antimicrobial activity of TLC eluted (spot 1) bioactive spot by agar well diffusion assay. **g** TLC analysis of acid hydrolyzed purified lipopeptide fraction (Lane 2) and intact lipopeptide (Lane 1) as compared to amino acids Glutamate, Leucine, Aspartate and isoleucine lane 3, 4, 5, and 6 respectively after visualization by ninhydrin using solvent system-I

The TLC analysis of lipopeptide extracted and column purified by various methods was done by using two solvent systems as described previously (Jha et al. 2016; Seghal Kiran et al. 2010). The TLC analysis results of chloroform/methanol extracted and HP-20 extracted lipopeptides from NB and MBM by chromatogram developed with solvent system-I, showed one yellow spot which is negative for ninhydrin with retardation factor (Rf) value 0.83 (Fig. 5a). Similarly, three spots were observed for iodine vapour and visible under UV (254) in TLC developed by solvent system-II with Rf value 0.89, 0.84, and 0.78 respectively for lipopeptide extracted by HP-20 from NB and MBM (Fig. 5b and c). The spot with Rf value 0.83 in solvent system-I and 0.89 in solvent system-II is considered as of the same lipopeptide which becomes white when sprayed with water. TLC analysis with solvent system II (Ethanol water 7:3) showed better resolution than system-I, hence further TLC analysis carry forward with solvent system-II for monitoring impurities in post chromatographic fractions. In conclusion, TLC analysis results showed more impurities in HP-20 extracted lipopeptide from NB as compared to chloroform, acid precipitated methanolic extract and from mineral base media, when sprayed with 0.2% Ninhydrin.

### Bioautography of TLC separated lipopeptides

In order to identify and confirm the bioactivity of TLC separated lipopeptides, TLC plate developed with solvent system-II, and were analyzed by bioautography using *B. cereus* NCIM 2703 as an indicator organism. Bioautography results showed only a spot with Rf value of 0.89 had the zone of inhibition against *B. cereus* NCIM 2703 (Fig. 5d). In order to confirm bioactivity of iodine positive spots, respective spots scraped from the TLC plate eluted with methanol and assessed for antimicrobial activity by well diffusion, the spot with Rf value 0.89 showed a 1.5 mm zone of inhibition against *B. cereus* NCIM 2703 (Fig. 5e and f) while other two spot lack antagonistic activity.

The cyclic and peptide nature of bioactive agent viz. lipopeptides was further confirmed by hydrolysis of TLC eluted peptide by 6 M HCL at 121 °C for 4 h. The results showed before hydrolysis no purple colour was observed with 0.2% ninhydrin (Fig. 5g Lane No. 1) while acid hydrolyzed fraction shows the development of purple colour confirms the cyclic nature of lipopeptide (Fig. 5g Lane No. 2). Developed spots corresponding to standard amino acid leucine and valine (Fig. 5g Lane No. 3, 4, 5, 6), which highlights cyclic lipopeptide might contain leucine and valine.

### Effect of temperature and pH on antibacterial lipopeptide and its solubility

**Fig. 6:**
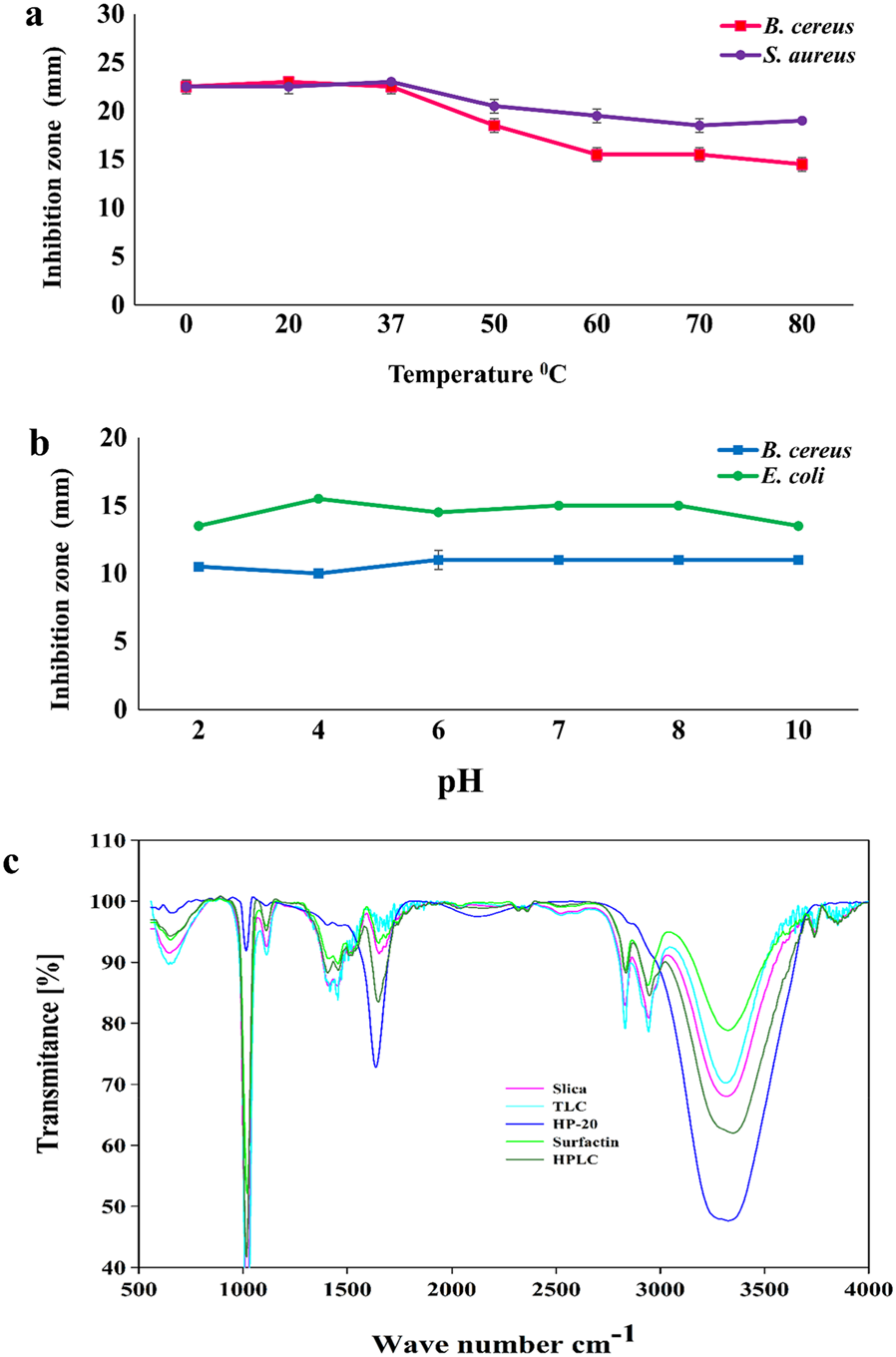
Effect of **a** Temperature and **b** pH on antimicrobial activity of surfactin lipopeptide against *B. cereus* NCIM 2703 (pink and blue respectively) and *S. aureus* NCIM 2654 (violet) and *E.coli* NCIM 2832 (green). **c** Fourier transform infrared spectroscopy of *B. velezensis* SK lipopeptide extracted by HP-20 (blue) and purified by TLC (cyan), HPLC (forest green), and Silica gel chromatography (pink)

Stability of extracted lipopeptide has been evaluated at various temperatures and pH with respect to its antagonistic activity, extracted lipopeptide showed thermo-stability over wide range of temperatures 0–80 °C. Specifically, lipopeptide exposed at temperatures 0 °C, 20 °C, and 37 °C retained maximum antimicrobial activity with a 24 mm zone of inhibition against *B. cereus* NCIM 2703 and *S. aureus* NCIM 2654 (Fig. 6a). Lipopeptides produced by *B. velezensis* SK showed pH stability over the range of 2 to 10, as the antimicrobial activity retained against *B. cereus* NCIM 2703 and *E. coli* NCIM 2832 (Fig. 6b). These results suggest that lipopeptide produced by *B. velezensis* SK exhibits good temperature (0-80 °C) and pH (2–10) stability and become potential applications in the biomedical and food industry. HPLC purified lipopeptide is soluble in 70% methanol, ethanol, and acetone, whereas partially soluble in distilled water and insoluble in chloroform and ethyl acetate.

### Fourier-transform infrared spectroscopy (FTIR) analysis of lipopeptides produced by *B. velezensis* SK

Chemical nature of bioactive compound i.e. lipopeptide produced by *B. velezensis* SK has been determined by the identification of functional groups present in the compound. FTIR spectra (Fig. 6c) exhibit strong absorption over 3240 to 3500 cm^−1^, with maxima at 3317 cm^−1^ which is due to –N–H, C–H, and –OH stretching, indicate the presence of carbon containing compound and with an amino group, absorption at this range also implies presence of intermolecular hydrogen bonds (Das et al. 2008). Presence of a sharp peak at 1647 cm^−1^ is indicative of the presence of amide group –C–N and carbonyl group stretching vibrations of protein and peptide (Jha et al. 2016; Sharma et al. 2018). The sharp absorption peak at 2825 cm^−1^ and 2939 cm^−1^ indicates the presence of a long aliphatic chain congaing –CH, –CH_2_, –CH_3_ such as the fatty acid chain of the lipopeptide. In addition, absorption peak at 1419 cm^−1^ and 1450 cm^−1^ might correspond to C–H alkyl bond vibration of aliphatic amino acids such as Leu, Ile, and absorption at 1112 cm^−1^ is due to vibration ester bond (Das et al. 2008; Kumar et al. 2009). Obtained spectra of purified lipopeptide from *B. velezensis* SK showed a similar type of bond stretching and vibration as standard surfactin (Sigma 98%). FTIR spectra of lipopeptides from *B. velezensis* SK also showed similarities with previously reported lipopeptides produced by *B. amyloliquefaciens* SAS-1 and *B. subtilis* BR-15 (Sharma et al. 2018). Hence, obtained results conclude that the antimicrobial peptide produced by *B. velezensis* SK is a cyclic lipopeptide containing a fatty acid chain and peptide backbone.

Lipopeptide extracted by Diaion HP-20 and CHCl_3_ shows smooth peaks, whereas TLC eluted, Silica gel fraction, and HPLC purified lipopeptides showed sharp peaks exactly similar to spectrum with standard surfactin (Fig. 6c and Additional file 1: Figure S7). FTIR analysis of lipopeptide extracted from cell-free culture media by methods described above also revealed the quality and purity of lipopeptides (surfactin).

### Identification and mass spectroscopic analysis of lipopeptides produced by *B. velezensis* SK (LC–ESI–MS)

Lipopeptide produced by *B. velezensis* SK purified by various methods further identified by LC–ESI–MS. LC–ESI–MS analysis of the partially purified HP-20 extract showed two clusters of peaks (Additional file 1: Figure S9a), ESI–MS analysis of peak at retention time (Rt) 46 to 56 min reveals a group of protonated molecular ion peak [M + H]^+^ of m/z ratio 1043.58, 1057.59, and 1071.61 indicating the presence of iturin homologues (C_14-16_), each of differs in the mass of 14 Da relative to –CH_2_–, which also influence its retention time with a marginal difference (Additional file 1: Figure S10 a, b and c; Table 2). Second cluster of peaks observed at the range of retention time 62 to 64 min in LC (Additional file 1: Figure S9a), further ESI–MS analysis of these cluster of peaks revealed protonated molecular ion peaks [M + H]^+^ of m/z ratio 994.67, 1008.69, 1022.70, and 1036.72 corresponds to four surfactin homologues C_12_-surfactin, C_13_-surfactin, C_14_-surfactin, and C_15_-surfactin respectively (Additional file 1: Figure S10d). Molecular ion peaks of lipopeptides obtained from *B. velezensis* SK in this study compare with average molecular ion mass of iturin and surfactin from literature (Dhanarajan et al. 2016; Goswami and Deka 2019; Souza et al. 2018; De Faria et al. 2011; Dang et al. 2019; Liu et al. 2020; Chen et al. 2020). LC–ESI–MS data of HP-20 extract suggest the *B. velezensis* SK mainly produce surfactin homologues with 76.75% relative abundance as compared to iturin 23.24% relative abundance (Table 2) in partially purified HP-20 extract. Further, within each family C_13_-Surfactin and C_14_-Iturin are found to be most abundant homologues (Table 2). Relative abundance is calculated by summing the major peak of all homologues (46 to 56 and 62 to 64 Rt) area and expressed as each family of lipopeptides as a percent value.

**Table 2:** LC–ESI–MS identification of surfactin and iturin type of homologues produced by *B. velezensis* SK*,* and comparison of their Molecular weight and peptide sequence reported in literature (Dhanarajan et al. 2016; Goswami and Deka 2019; Souza et al. 2018; De Faria et al. 2011; Dang et al. 2019; Liu et al. 2020; Chen et al. 2020)

RT (min)	Observed MW [M + H]^+^ (m/z)	Average MW reported	Identified lipopeptide	Relative % of lipopeptide family	Amino acid sequence (Obtained from literature)
HP-20 Extract					
Peak first					
45.89	1043.58	1042.7	C_14_-Iturin	11.37	Cyclic (FA-N-Y-N-Q-P-N-S)
49.97	1057.59	1057.6	C_15-_Iturin	5.00	Cyclic (FA-N-Y-N-Q-P-N-S)
55.33	1071.61	1071.7	C_16-_Iturin	6.86	Cyclic (FA-N-Y-N-Q-P-N-S)
Peak second					
63.52	994.67	994.21	C_12_-Surfactin	17.49	Cyclic (FA-E-L/I-L/I-V- L/I- D-L)
	1008.69	1008.87	C_13_-Surfactin	27.73	Cyclic (FA-E-L/I-L/I-V- L/I -D-L)
	1022.70	1023.18	C_14_-Surfactin	19.11	Cyclic (FA-E-L/I-L/I-V-V-D-L)
	1036.72	1036.78	C_15_ Surfactin	12.40	Cyclic (FA-E-L/I-L/I-V- L/I -D-L)
Fraction 2 (40% Acetone pH 4)					
Peak first					
45.88	1043.58	1042.7	C_14_-Iturin	0.71	Cyclic (FA-N-Y-N-Q-P-N-S)
50.48	1057.59	1057.6	C_15_-Iturin	0.20	Cyclic (FA-N-Y-N-Q-P-N-S)
Peak second					
62.42	1036.72		C_15_-Surfactin	44.34	Cyclic (FA-E-L/I-L/I-V-D -L/I-L)
63.54	994.67	994.21	C_12_-Surfactin	54.74	Cyclic (FA-E-L/I-L/I-V-D-L/I-L)
	1008.69	1008.87	C_13_-Surfactin		Cyclic (FA-E-L/I-L/I-V-D-L/I-L)
	1022.70	1023.18	C_14_-Surfactin		Cyclic (FA-E-L/I-L/I-V-D-L/I-L)
	1036.72	1036.78	C_15_-Surfactin		Cyclic (FA-E-L/I-L/I-V-D-L/I-L)
	1050.73	1050.92	C_16_-Surfactin		Cyclic (FA-E-L/I-L/I-V-D-L/I-L)
Fraction 5 (80% Acetone pH 8)					
62.20	994.67	994.21	C_12_-Surfactin	24.17	Cyclic (FA-E-L/I-L/I-V-D-L/I-L)
63.38	1022.70	1023.18	C_14_-Surfactin	32.18	Cyclic (FA-E-L/I-L/I-V-D-L/I-L)
	1036.72	1036.78	C_15_-Surfactin	43.64	Cyclic (FA-E-L/I-L/I-V-D-L/I-L)

FA; Fatty acid, N: Asparagine; Y: Tyrosine; Q: Glutamine; P: Proline; S: Serine; E: Glutamic acid; L: Leucine; I: Isoleucine; V: Valine; D: Aspartic acid

LC–ESI–MS analysis of HP-20 dual gradient fractions (F2 and F5) showed separation of iturin in fraction 2 (25% acetone) which shows two peaks (Additional file 1: Figure S9b), one around retention time of 45–50 and other at range of 62 to 64, which is similar to mass and retention time observed in LC–MS analysis of partially purified HP-20 extract. The ESI–MS analysis confirms the presence of negligible amount of iturin (relative abundance 0.91%) with molecular ion peaks [M + H]^+^ 1043.58 and 1057.59 (Additional file 1: Figure S11a and b) and most abundant surfactin (99.08%) with its homologues having m/z ratio [M + H]^+^ 994.67, 1008.69, 1022.70, 1036.72 (most abundant 44.34%) and 1050.73 (Additional file 1: Figure S11c, d). In detail, Rt of peaks detected in all fractions along with their relative abundance and assignment of lipopeptides homologues were shown in Table 2.

**Fig. 7:**
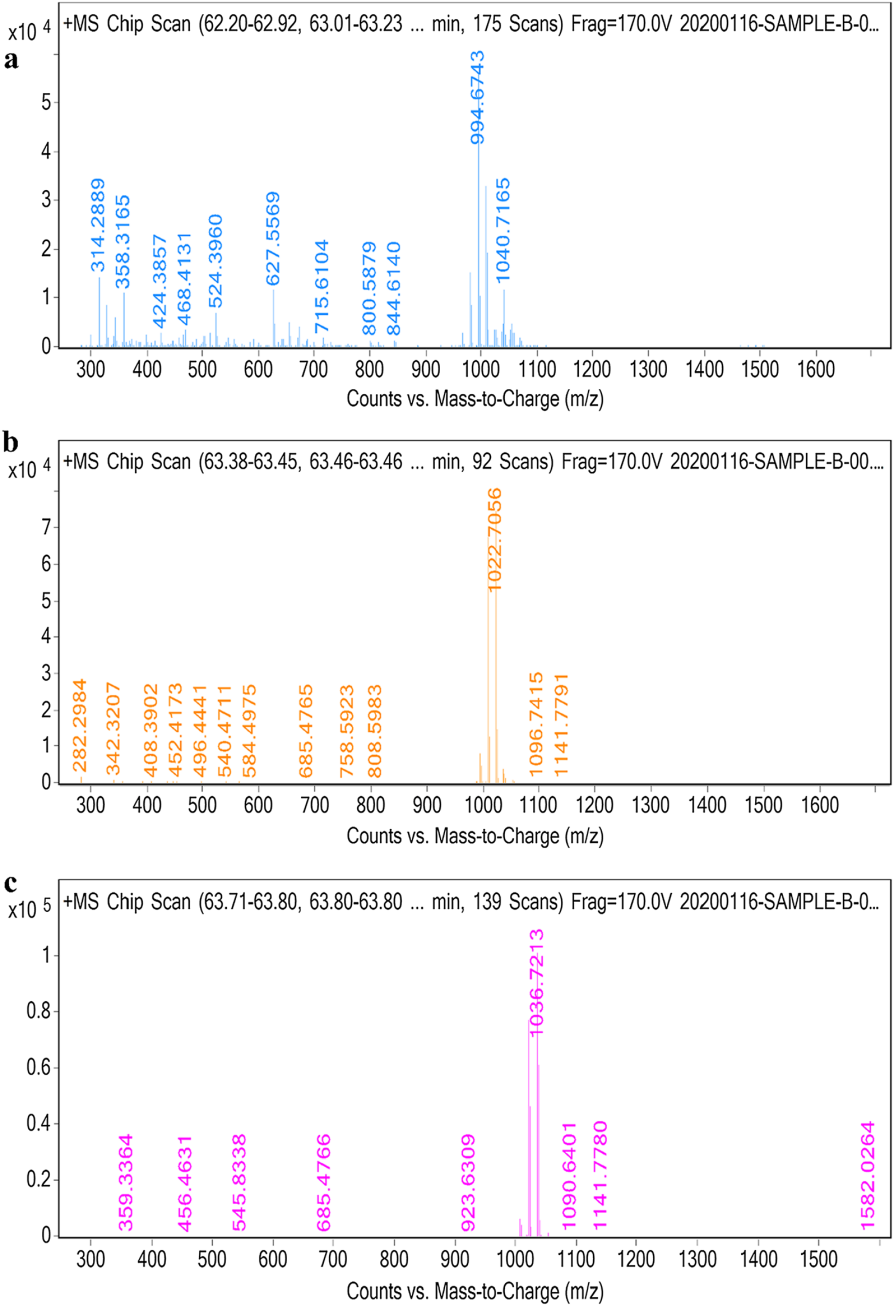
Full ESI–MS scan of LC separated peak from dual gradient fraction (F5 80% acetone pH8) in positive ion mode showing molecular ion [M + H] of peaks at range of 62 to 64 min **a** C_12_-Surfactin **b** C_14_-Surfactin **c** C_15_-Surfactin

LC–ESI–MS analysis of fraction 5 (F5) showed a major peak at 63 min (Figure S9c) with molecular ion peak of m/z 1036.72 (43.64% relative abundant) correspond to most abundant homologues C_15_-surfactin (Fig. 7a, b, and c). A similar type of scenario was observed with HPLC purified surfactin lipopeptides with 80% methanol. Overall, LC–ESI–MS analysis of HP-20 extract and dual gradient chromatography fractions (F2 and F5) confirmed the separation of iturin in fraction 2 from fraction 5 (consist most abundant surfactin isoforms), hence we conclude that the *B. velezensis* SK mainly produce surfactin type of lipopeptide.

### Antimicrobial activity of lipopeptide

Indicator organisms for antimicrobial activity include streptomycin-resistant *B. cereus* ATCC 10,876 and opportunistic pathogens such as *S. aureus* NCIM 2654 and methicillin-resistant *S. aureus* (MRSA). Surfactin lipopeptide showed the highest antimicrobial activity by spot on lawn method against non-resistant *B. cereus* NCIM 2703 with 32,000 AU/ml and six months stored extract (aliquot) with 16,000 AU/ml reflecting its stability during storage (Table 3). Similarly, both partially purified surfactin and surfactin stored at 4 °C showed a similar degree of inhibitory activity at 16,000 AU/ml against streptomycin-resistant *B. cereus* ATCC 10,876 and *S. aureus* NCIM 2654, while stored aliquot with lesser activity 400 AU/ml. The antagonistic activity was also tested against methicillin-resistant *S. aureus*; results showed inhibitory activity with 800 AU/ml slightly more as compared to stored lipopeptide 400 AU/ml. Our results including agar well diffusion assay showed the potent and broad-spectrum antimicrobial activity against Gram-positive including drug-resistant and Gram-negative organisms. Hence, partially purified lipopeptide could be useful to control drug-resistant organisms present in the hospital environment as a disinfectant, as stated earlier study by Singh et al. which demonstrated the disinfectant-like activity of lipopeptides produced by *B. tequilensis strain* SDS21 (Singh and Sharma 2020).

**Table 3:** Antimicrobial activity of purified surfactin against selected Gram-positive including antibiotic-resistant food born pathogen

Indicator Strains	Antimicrobial activity (AU/ml)	
	Lipopeptide	Lipopeptide*
*B. cereus NCIM* 2703	32,000	16,000
*B. cereus ATCC* 10,876	16,000	16,000
*S. aureus NCIM* 2654	16,000	200
*S. aureus (MRSA)*	800	400

## Discussion

AMPs could be an alternatives for the conventional antibiotics to which microorganisms especially opportunistic pathogens developed resistance and thus essential to control (Alekshun et al. 2007; Hassan et al. 2007; Nadaf et al. 2018). To overcome the resistance problem, present study was aimed to search for potent AMPs producing microorganisms. In primary screening, we isolated and identified a new potent antimicrobial lipopeptide producing strain *B. velezensis* SK by genotypic, morphologically characterization, as it displays broad-spectrum antimicrobial activity against both Gram-positive and Gram-negative bacteria including drug-resistant opportunistic pathogens.

Further, lipopeptide production was evaluated in various media and results showed isolated *B. velezensis* SK could able to produce lipopeptides in all media tested including MBM. Earlier, *B. velezensis* and related species have been shown to produce lipopeptides (0.818 gL^−1^) from different agro-industrial wastes and office waste such as paper hydrolyzed by *B. velezensis* ASN1 which can be used in the production medium to lower the cost of production (Jha et al., 2016; Nair et al. 2020). Time course and lipopeptide production kinetic experiments with MBM revealed that CuSO_4_ completely inhibits the production of lipopeptide by *B. velezensis* SK*,* while the presence of Zn^2+^ is unaffected. Earlier studies highlighted that these trace elements along with Co^2+^, and Ni^2+^ inhibit the growth and lipopeptide production of *B. subtilis* (Rangarajan and Clarke 2015). The ESI–MS analysis of the crude extract and purified fractions receives attention as it highlights the presence of surfactin and its isoforms as a major constituent (Table 2), although several earlier studies report the production of three types of lipopeptides from *Bacillus Spp*. (Iturin, fengycin, and surfactin) with similar cultural conditions within 24–72 h and mainly reported for antifungal activity (Chen et al. 2020; Li et al. 2020a, b; Liu et al. 2020; Sarwar et al. 2018), interestingly *B. velezensis* SK with culture conditions (37 °C, 120 rpm in 48 h) in this study mainly found for antibacterial surfactin production. However, earlier studies based on genome analysis have been reported for several antagonistic agents from *Bacillus velezensis* and *Bacillus Spp*. (Grady et al. 2019; Li et al. 2020a, b; Li et al. 2020a, b; Palazzini et al. 2016; Wu et al. 2016), hence presented culture conditions and subsequent extraction of antimicrobial with HP-20 could be useful for selective surfactin production and extraction, purification.

Most previous studies reported acid precipitation for lipopeptides isolation from a variety of bacterial strains (De Faria et al. 2011; Das et al. 2008; Rangarajan and Clarke 2016; Sarwar et al. 2018; Singh and Sharma 2020). Present study also focuses on comparative lipopeptide extraction methods for an easy and convenient extraction process, which found that the lipopeptides extracted better with HP-20 Diaion resin from culture medium with remarkable antimicrobial activity against a broad range of bacteria among other extraction methods.

Comparative chromatographic techniques have been used for the purification of lipopeptides from the partially purified extract. Silica gel, size exclusion column chromatography method could not separate families of lipopeptides (Iturin or surfactin). Hence, an individual family of surfactin and iturin was purified at homogeneity by using dual gradient chromatography and identified by the RP-HPLC method. The families of lipopeptides and their isoforms produced by *Bacillus spp*. can be poorly separated by using only solvent gradients such as acetone or methanol due to their similar polarity (Dhanarajan et al. 2015; Zhao et al. 2016). The TLC, FTIR, and biochemical characterization of the antimicrobial agent from *B. velezensis* SK identified amphipathic, pH, and thermo-stable cyclic lipopeptides (Detail physiochemical character mention in Additional file 1: Table S6). In addition, FTIR analysis of various partial purified and purified fractions with surfactin revealed the purity of surfactin, when compared to standard surfactin. However, the antimicrobial activity of purified lipopeptide did not affect by organics solvents (methanol, ethanol, acetone, chloroform, and ethyl acetate as used during purification), which indicates their solvent stability (Additional file 1: Table S6). Further, *in-vitro* bioautography of TLC separated surfactin confirmed its antagonistic activity against food-born pathogen *B. cereus* NCIM 2703. Altogether, antimicrobial activity results by various methods confirmed that the HP-20 crude extract and purified surfactin could inhibit the growth of *E.coli*, *P. vulgaris*, *S. aureus, and B. cereus* including streptomycin-resistant strains. LC–ESI–MS analysis of partially purified HP-20 extract and dual gradient purified fractions showed *B. velezensis* SK mainly produces a surfactin group of lipopeptides with a small amount of iturin. *B. velezensis* SK surfactin mainly comprises five homologues of varying β-hydroxy fatty acid with most abundant C_15_-Surfactin (m/z 1036).

In summary, this study resulted in the isolation of potent antibacterial surfactin producing new strain *B. velezensis* SK. The new strain and its concomitant antimicrobial potential of surfactin production could be useful to combat drug-resistant human pathogens and food-born pathogen *B.cereus* ATCC 10,876, responsible to cause diseases such as bacteremia, pneumonia in immune-compromised patients. Similarly, isolated *B. velezensis* SK strain could be a used as probiotic, a similar type of *B. velezensis* CPA1-1 strain has been reported as a probiotic in aquaculture industries (Li et al., 2020a, b). Thus, the isolated lipopeptide producer strain could be useful at a commercial level due to its potential to produce lipopeptide.

## Supplementary Information

**Additional file 1.** Additional datasets of figures and tables supporting conclusions of this article includes in additional information.

## Data Availability

All relevant data are within the paper and its additional information files, and 16 s rRNA data have been deposited in “GenBank” with the accession codes MK007327.1 are fully available.
