# Supplementary material for: Purification and characterization of antibacterial surfactin isoforms produced by Bacillus velezensis SK
Source: AMB Express. 2022 Jan 27;12:7. doi: 10.1186/s13568-022-01348-3 (PMC8795249; doi:10.1186/s13568-022-01348-3)
Supplement: Supplementary file 1 — Additional file 1. Additional datasets of figures and tables supporting conclusions of this article includes in additional information. [file 13568_2022_1348_MOESM1_ESM.docx]

**AMB Express**

**Purification and characterization of antibacterial surfactin isoforms produced by *Bacillus velezensis* SK.**

**(Supplementary material)**

Sagar S. Barale^1^, S. G Ghane^3^ Kailas D. Sonawane^1,2*^

^1^ Department of Microbiology, Shivaji University, Kolhapur 416004, Maharashtra (MS), India.

^2^ Structural Bioinformatics Unit, Department of Biochemistry, Shivaji University, Kolhapur 416004, Maharashtra (M.S.), India.

^3^ Department of Botany, Shivaji University, Kolhapur 416004, Maharashtra (M.S), India.

*Corresponding author

Prof. Kailas Dasharath Sonawane, Ph.D.

Structural Bioinformatics Unit,

Department of Biochemistry,

Shivaji University, Kolhapur 416 004,

Maharashtra (M.S.), India.

Phone: +91 9881320719, +91 231 2609153

Fax No. : +91 231 2692333

Email: [kds_biochem@unishivaji.ac.in](mailto:kds_biochem@unishivaji.ac.in)

**Supplementary Figure**

**
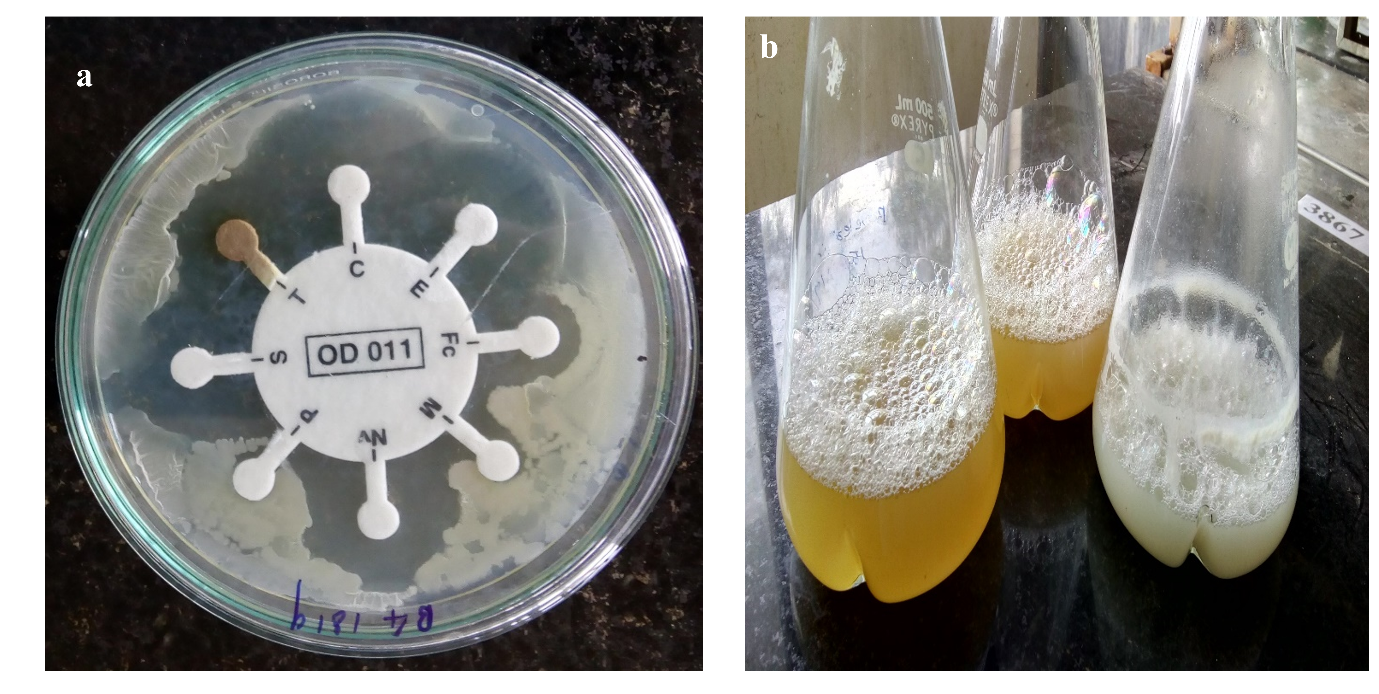
**

**Figure S1** Antibiotic suceptiblity of *B. velezensis* SK against selected antibiotics (A) and production of stable foam in nutrient broth (right) and in MBM (leaft) highlights lipopeptides production.


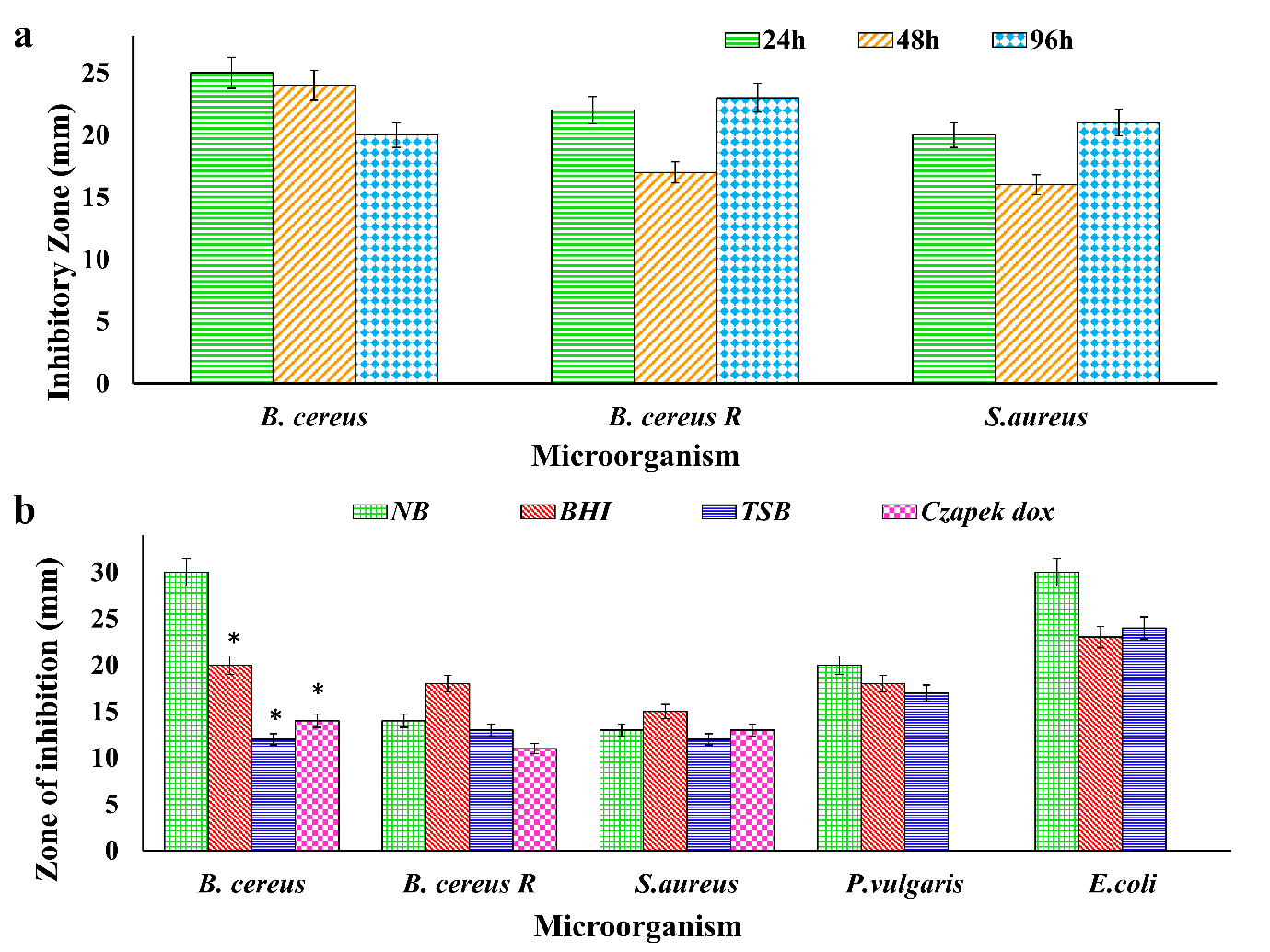


**Fig. S2** Antimicrobial activity of lipopeptide produced by *B. velezensis* SK against panel of bacteria (A) At various incubation time in NB, and (B) In various culture media.


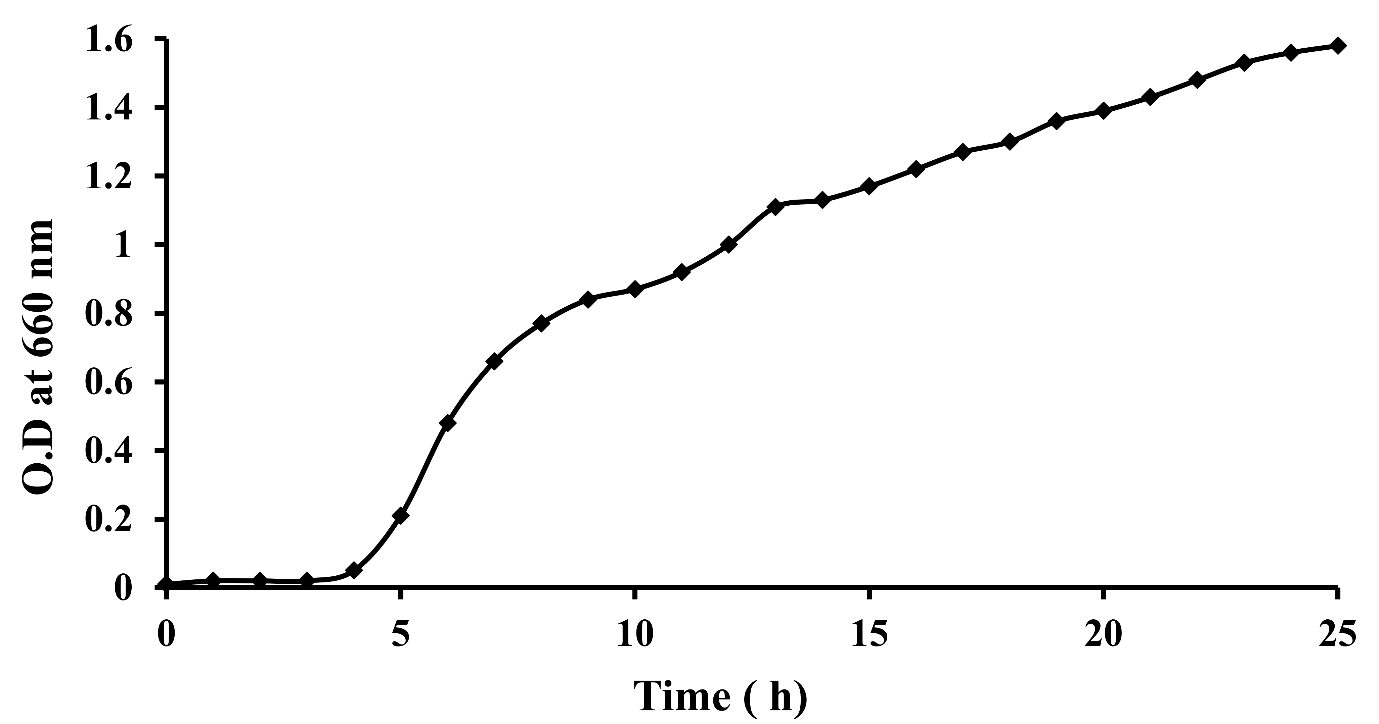
 **Figure S3** Growth curve analysis of *B. velezensis* SK in nutrient broth at 370C, 120 rmp agitation for 24 h.


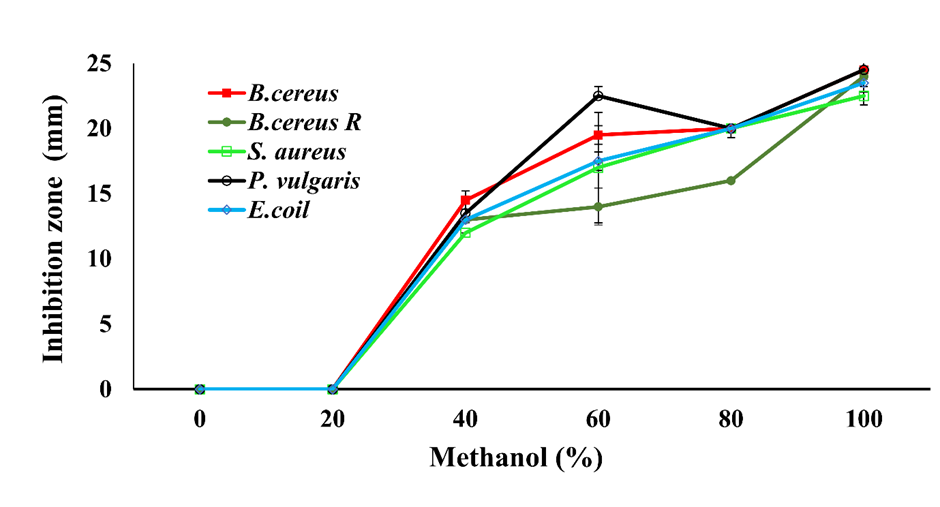


**Figure S4** Antimicrobial activity of six fractions against panel of indicator organism for optimization of Diaion HP-20 extraction method with decreasing polarity of methanol.


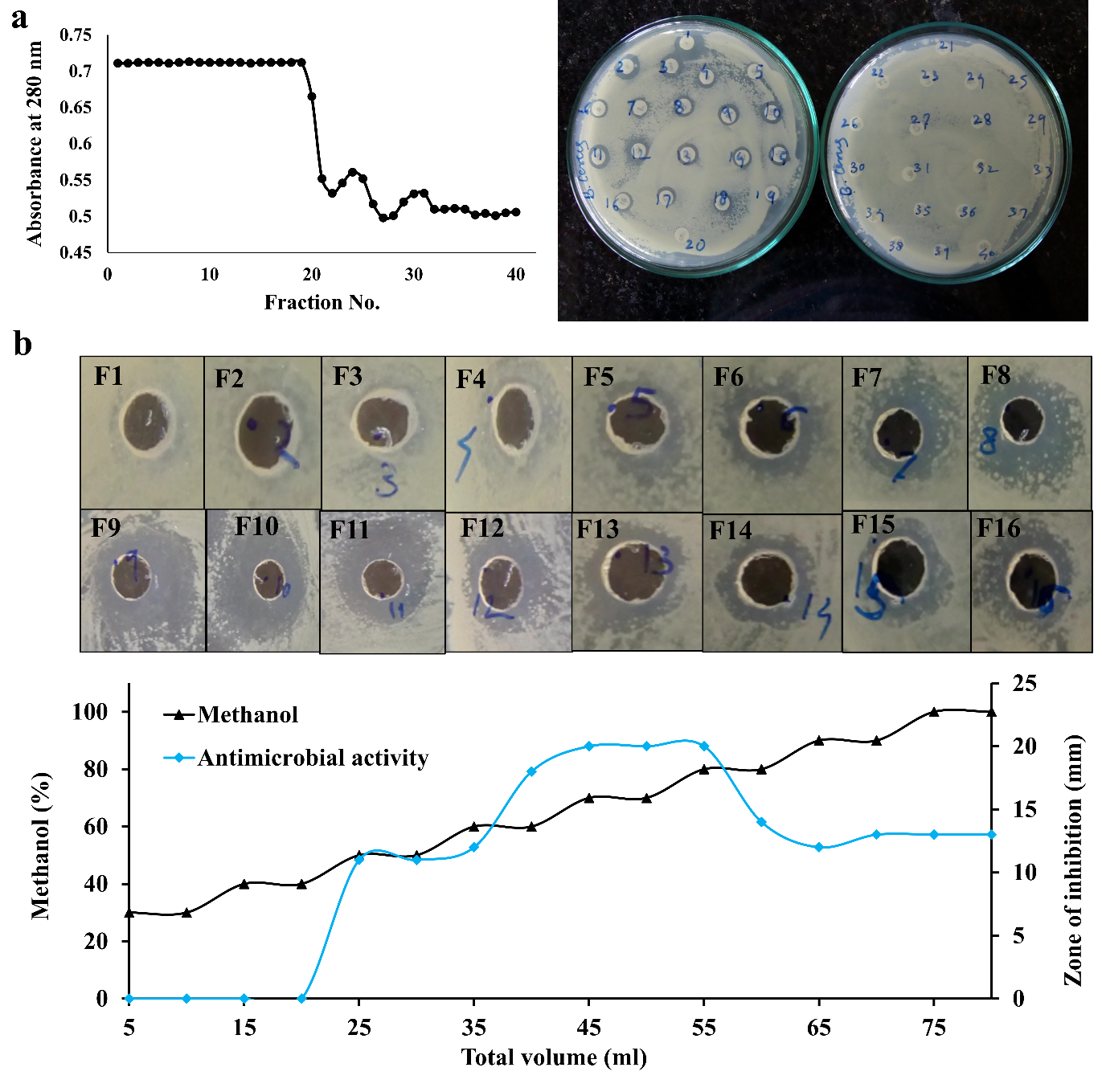


**Figure S5** Silica gel column chromatography purification of lipopeptides using two solvent systems **(a)** Chloroform: Methanol gradient fractions (40) with UV-absorption spectra at 280 nm, and their antimicrobial activity against *B. cereus* NCIM 2703 by paper disk method **(b)** Methanol gradient (30% to 100%) with antimicrobial activity of column purified fractions (16, upper panel) against *B. cereus* NCIM 2703 by agar well diffusion assay and antagonistic activity in inhibitory zone (diameter in mm) corresponds solvent gradient.


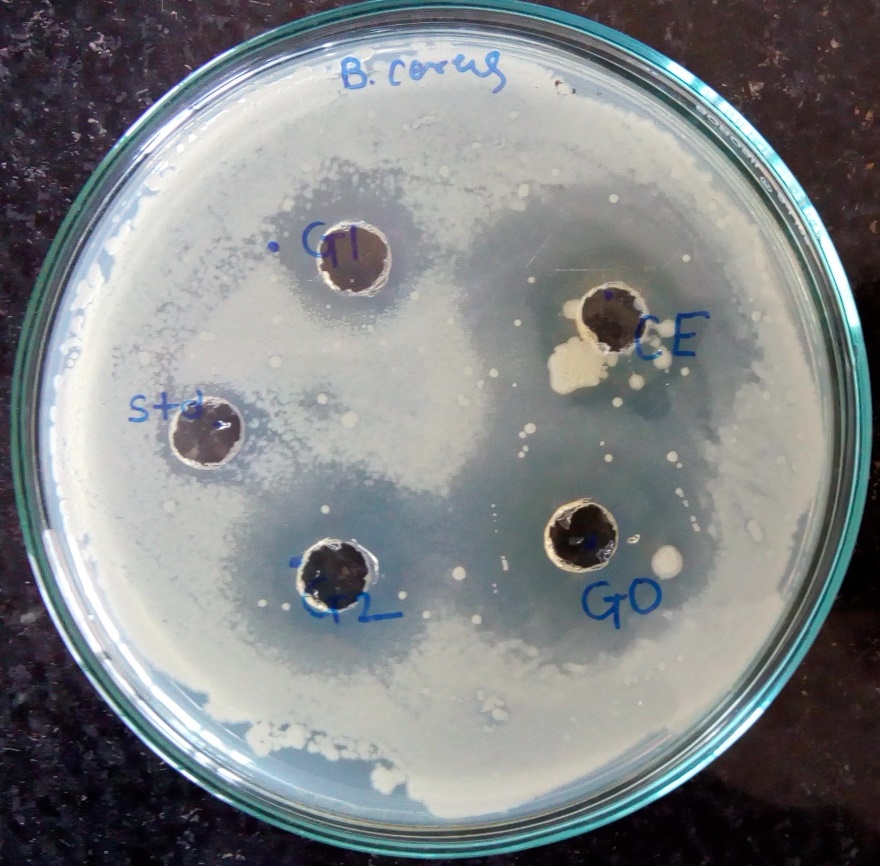


**Figure S6** Antimicrobial activity of selected Sephadex LH-20 column fractions against *B. cereus* NCIM 2703.


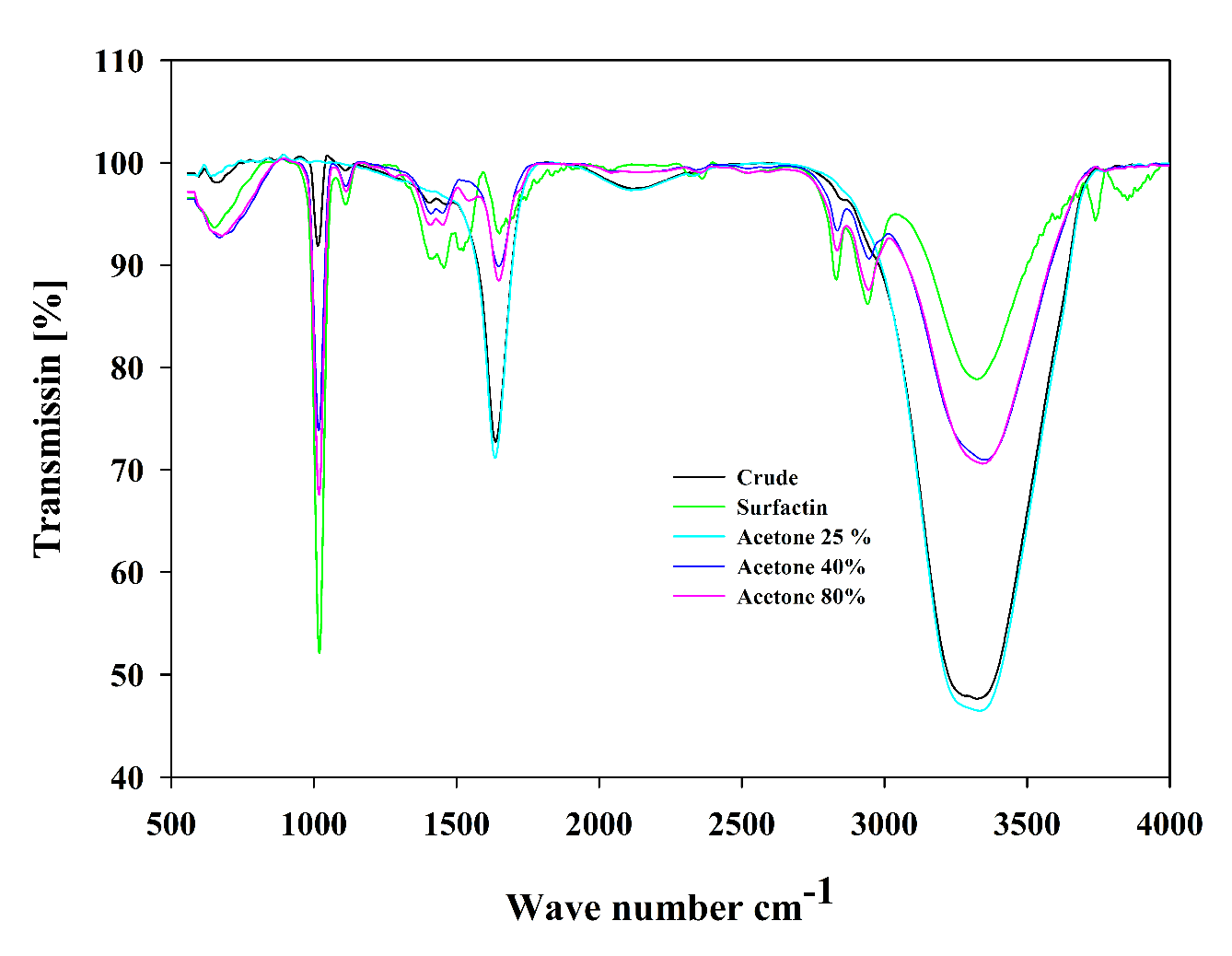


**Figure S7** FTIR spectra of lipopeptide purified fractions from HP-20 dual gradient chromatography.

**
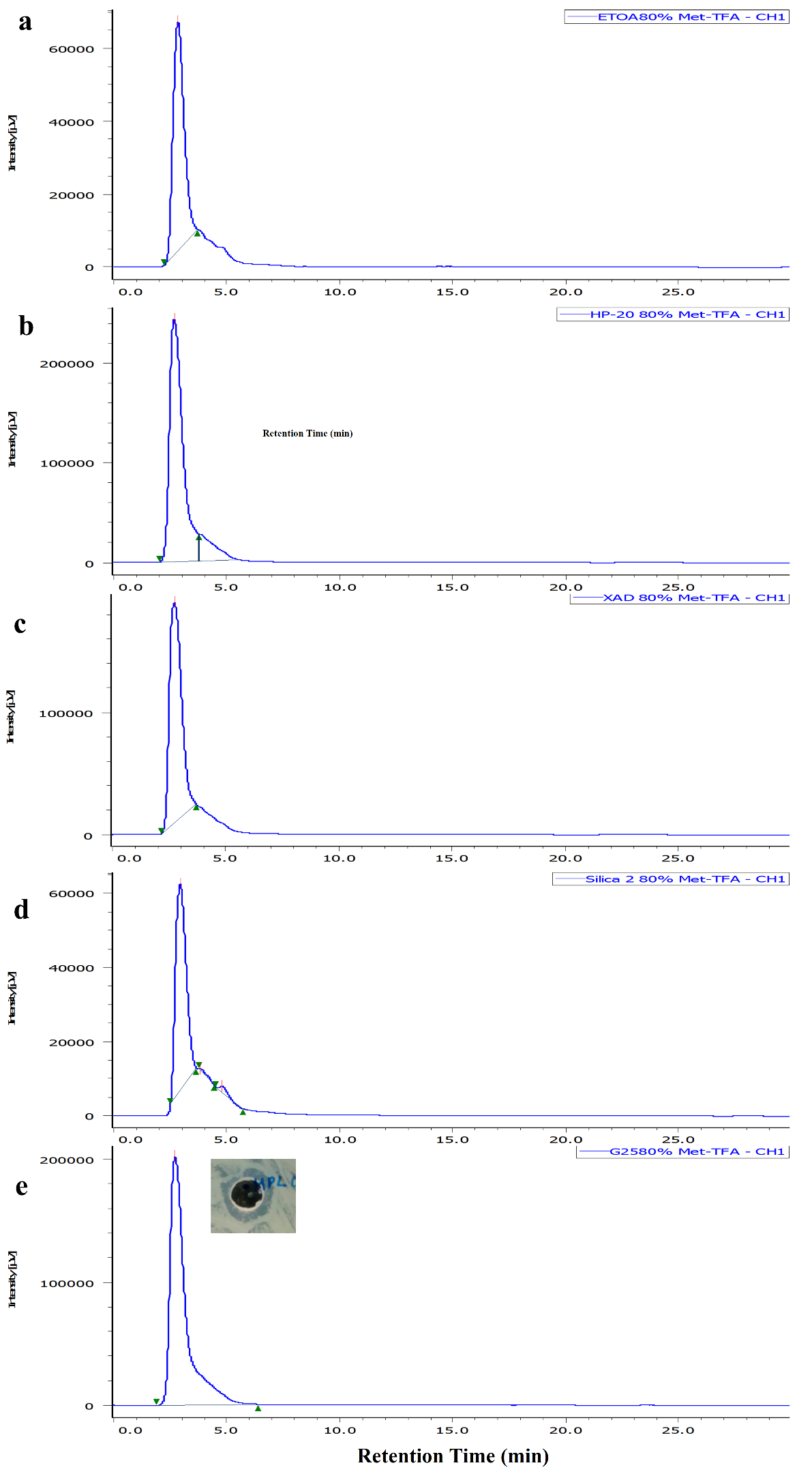
**

**Figure S8** RP-HPLC analysis of lipopeptide fractions purified by (a) Ethyl acetate (b) Diaion HP-20 (c) Amberlite XAD-16 purified extract (d) Silica gel column chromatography (e) Sephadex LH-20 chromatography by using mobile phase 80% methanol in water.

**
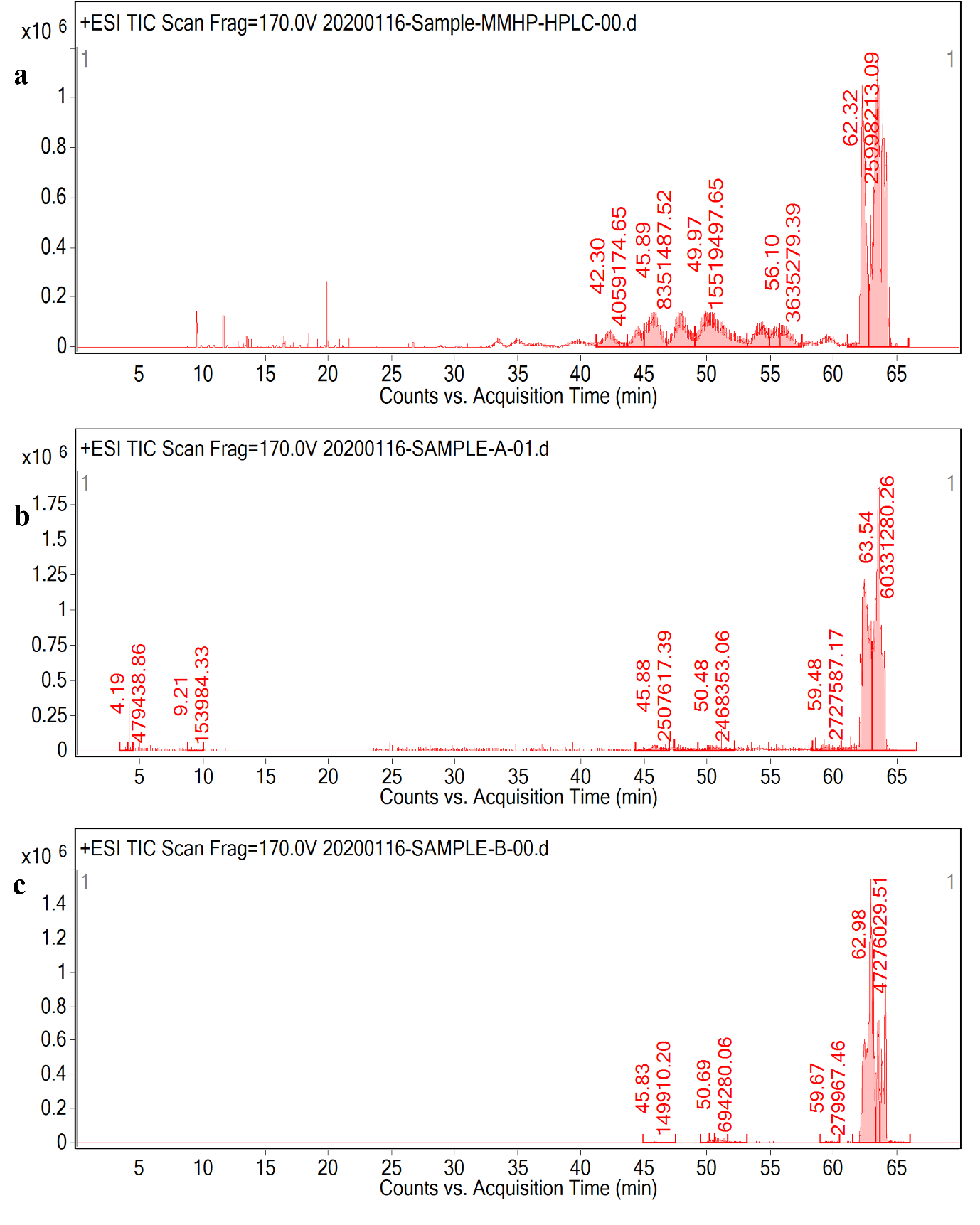
**

**Figure S9** Total ion chromatogram (TIC) from LC-MS, of partially purified lipopeptides from *B. velezensis* SK (a) and lipopeptide purified by dual gradient as (b) Acetone 25% pH 4(F2) (c) Acetone 80% pH 8(F5).

**
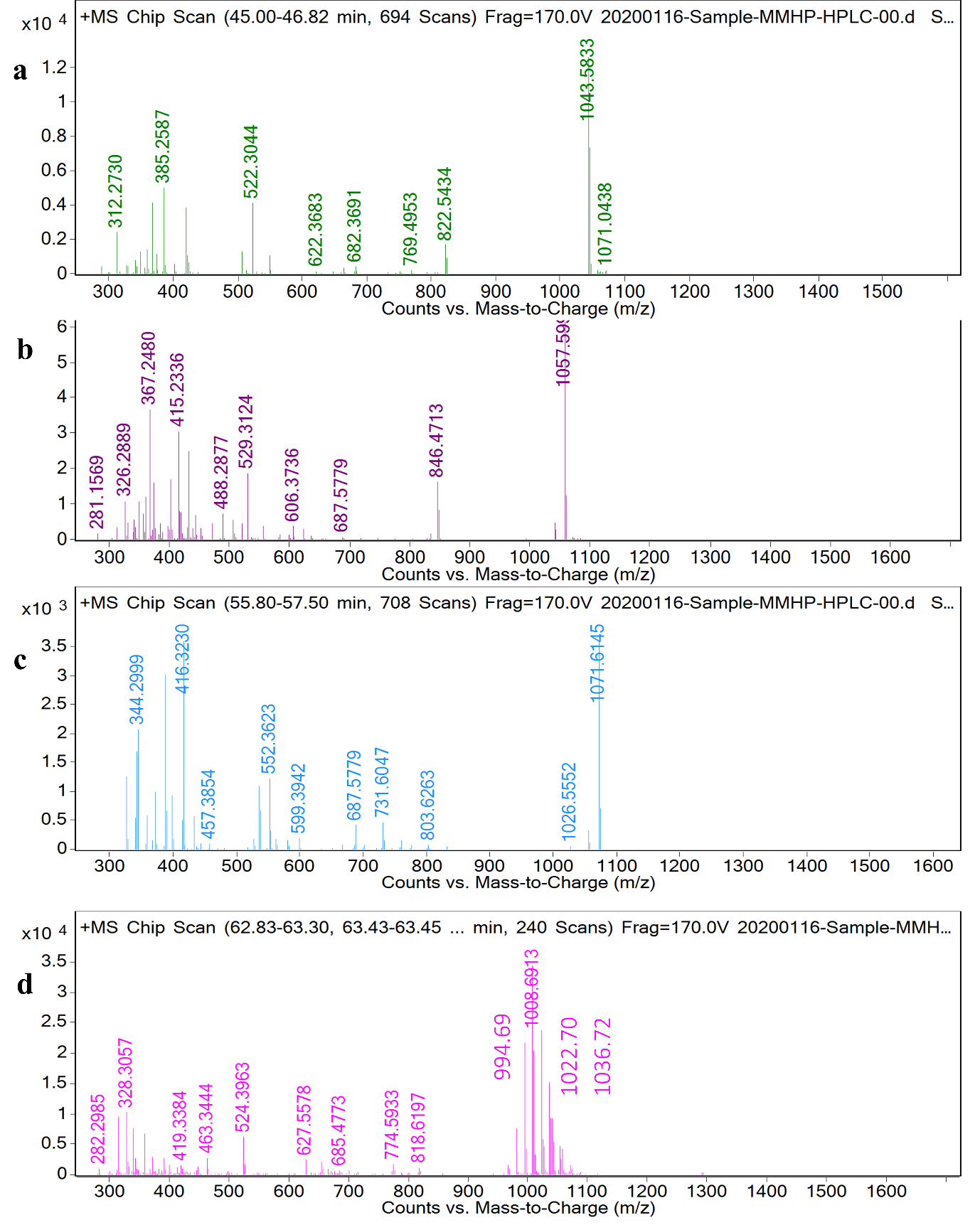
**

**Figure S10** Full ESI-MS scan of partial purified and LC separated lipopeptides in positive ion mode showing molecular ion peak [M+H] of (a) Peak at retention time 45.89 min C_14_-Iturin (b) C_15_-Iturin at 49.97 min RT (c) C_16_-Iturin at 55.8 min RT and (d) Cluster of peak at 63.52 min RT for surfactin homologues.


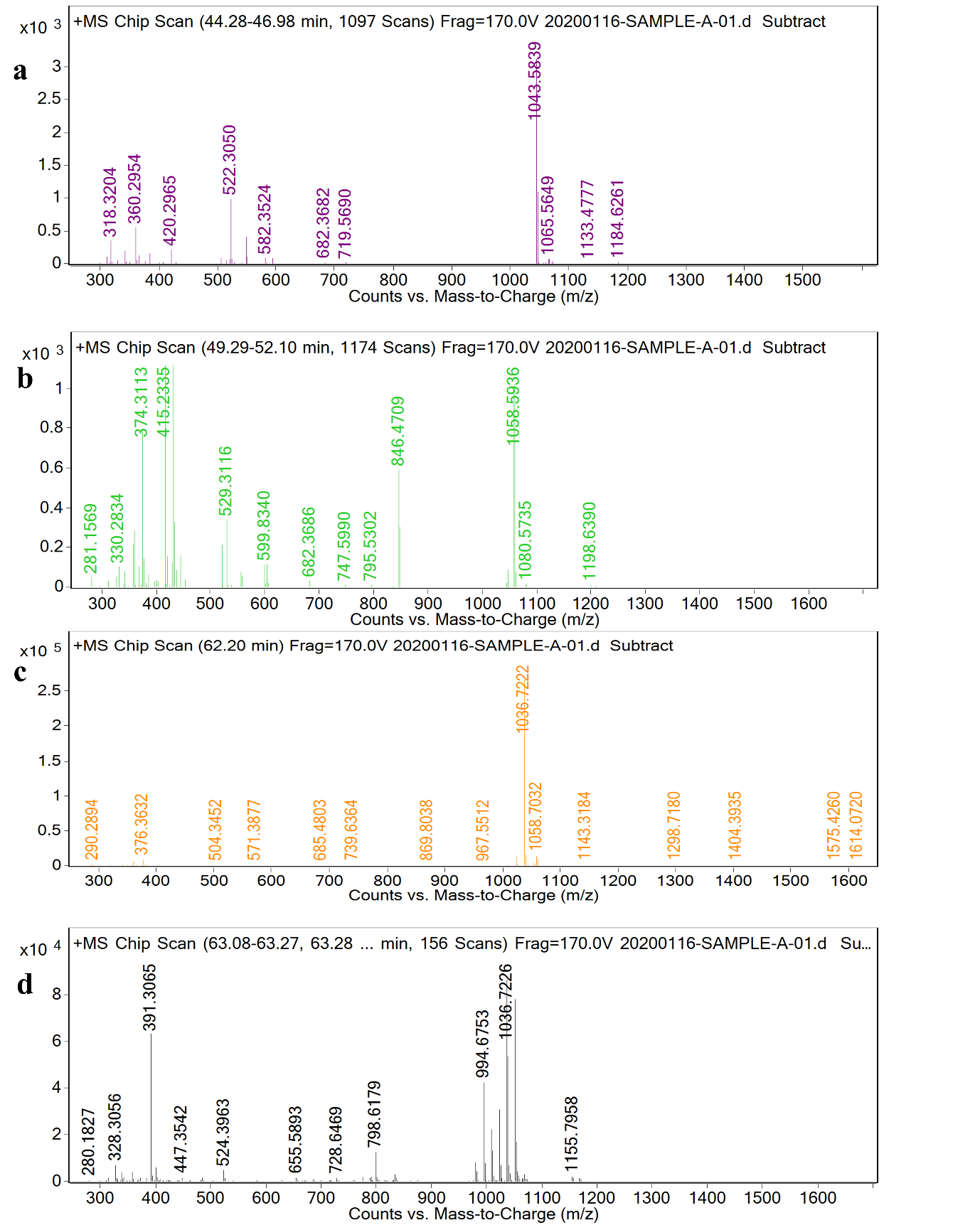


**Figure S11** Full ESI-MS scan of LC separated peak from dual gradient fractions (F2 25% acetone pH 4) in positive ion mode showing molecular ion [M+H] (a) ESI-MS spectra of peaks at range of 45 to 51 min for C14-Iturin homologues (b) C15- Iturin and (c) Peak around 62.20 min RT correspond C15-Surfactin homologue (D) C14-Surfactin, C15-Surfactin, and C16-Surfactin.

**Supplementary tables:**

**Table S1** Physiological properties of resins.

| Resin | Polarity | Surface area (m^2^/g) | Pore volume (mL/g) | Mean pore size (nm) |
| --- | --- | --- | --- | --- |
| XAD-16 | Non-polar | 800 | 0.55 | 20 |
| HP-20 | Non-polar | 500 | 1.3 | 26 |

**Table S2** Dual gradient elution program for lipopeptide separation.

| Fraction No. | Solvent % (ml) | pH | Faction Volume of (ml) |
| --- | --- | --- | --- |
| F1 | 0 | NA | 250 |
| F2 | 25 | NA | 75 |
| F3 | 40 | 4 | 100 |
| F4 | 65 | 4 | 100 |
| F5 | 80 | 6 | 75 |
| F6 | 95 | 8 | 125 |
| F7 | 100 | 8 | 50 |

**Table S3** Primary screening of AMP producer by spot on lawn method against indicator microorganism.

| Isolate Identity | *B.cereus* | *B.cereus R* | *B. subtilis* | *S.aureus* | *P. aeruginosa* | *S. typhimurium* | *E.coli* |
| --- | --- | --- | --- | --- | --- | --- | --- |
| **A**1 | +++ | ++ | + | - | - | - | - |
| **A** 8 | ++ | - | - | - | - | - | - |
| **A**9 | + | - | - | - | - | - | - |
| **A**15 | +++ | - | - | - | - | + | + |
| **A19** | - | - | - | + | + | - | ++ |
| **A**21 | ++ | ++ | + | - | - | - | - |
| **A**23 | ++ |  | - | - | - | - | + |
| **A**35 | +++ | ++ | ++ | - | - | - | - |
| **A**38 | + | - | + |  | - | + | - |
| **A**47 | + | + | - | + | - | + | - |
| **B**4 | +++ | +++ | + | +++ | + | + | + |
| **B19** | + | + | - | + | - | - | - |
| **B23** | ++ | ++ | + | - | - | - | - |
| **C2** | + | + | - | - | - | + | + |
| **C3** | - | - | - | - | - | - | + |
| **C5** | ++ | ++ | - | - | - | + | - |
| **C12** | +++ | ++ | - | + | - | - | + |

**Table S4** Characteristics of *B. velezensis* SK in comparison of related species from literature.

| Characteristics | *B. velezensis* SK | *B. velezensis CPA1-1* | *B. velezensis BM 21* | *B. vallismortis DSM11031* | *B. velezensis CBMB205* | *B. amyloliquefaciens SYBC H47* |
| --- | --- | --- | --- | --- | --- | --- |
| Pigmentation | Creamy white | Dirty white | Milky white | Dark brown | Creamy white | Creamy white |
| Sucrose | + Ve | ND | +Ve | ND | + Ve | + Ve |
| Lactose | - Ve | + | + Ve | - | + Ve | + Ve |
| Xylose | - Ve | ND | - Ve | ND | - Ve | ND |
| ONPG | - Ve | + | ND | + Ve | ND | - Ve |
| Citrate | - Ve | ND | ND | ND | - Ve | + Ve |
| Voges-Proskauer | + Ve | ND | + Ve | ND | + Ve | + Ve |
| Catalase | + Ve | ND | + Ve | ND | + Ve | + Ve |
| Nitrate reduction | + Ve | ND | - Ve | ND | + Ve | + Ve |
| Indol | - Ve | ND | ND | ND | - Ve | + Ve |

Note; ND, Not determine, +Ve, positive, -Ve, Negative.

**Table S5** Antibiotic resistivity of isolated AMP producer strain against standard antibiotics.

| Antibiotics | Zone of Inhibition ± SD (mm) |
| --- | --- |
| Chloramphenicol | 34.5± 0.2 |
| Erythromycin | 28.5± 0.07 |
| Fusidic acid | 13± 0.07 |
| Methicillin | 0± 0.00 |
| Novobiocin | 24 ± 0.00 |
| Penicillin G | 0 ± 0.00 |
| Streptomycin | 23 ± 0.07 |
| Tetracycline | 20 ± 00.07 |

**Table S6** Physiochemical properties of surfactin lipopeptide from *B. velezensis SK*.

| **Physiological property of lipopeptide antibiotic** | **Observation** |
| --- | --- |
| Appearance | Yellowish |
| [M+H]^+^ m/z | 994.67, 1008.69,1022.70,1036.72 (abundant),1050.73 |
| Molecular formula | C_53_H_93_N_7_O_13_ (1036.72 C_15_-Surfactant most abundant) |
| UV λ_max_ | 280 nm |
| Colour test | Ninhydrin negative, Bradford positive, iodine vapour positive |
| Solubility | Soluble in 70% methanol, ethanol, acetone  Insoluble in chloroform, ethyl acetate, water |
| FTIR spectra (Wavenumber) | 3317 cm^-1^ , 1647 cm^-1^, 2825 cm^-1^ and 2939 cm^-1^, 1419 cm^-1^ and 1450 cm^-1^ |
| TCL Rf value | 0.89 |
